# Machine intelligence-driven framework for optimized hit selection in virtual screening

**DOI:** 10.1186/s13321-022-00630-7

**Published:** 2022-07-22

**Authors:** Neeraj Kumar, Vishal Acharya

**Affiliations:** 1 Functional Genomics and Complex System Lab, Biotechnology Division,The Himalayan Centre for High-throughput Computational Biology (HiCHiCoB, A BIC Supported by DBT, India), CSIR-Institute of Himalayan Bioresource Technology, Palampur, 176061 Himachal Pradesh India; 2 Academy of Scientific and Innovative Research (AcSIR), Ghaziabad, 201002 India

**Keywords:** Virtual screening protocol, Machine-learning, Deep learning, Instance-based learning, Lead optimization

## Abstract

Virtual screening (VS) aids in prioritizing unknown bio-interactions between compounds and protein targets for empirical drug discovery. In standard VS exercise, roughly 10% of top-ranked molecules exhibit activity when examined in biochemical assays, which accounts for many false positive hits, making it an arduous task. Attempts for conquering false-hit rates were developed through either ligand-based or structure-based VS separately; however, nonetheless performed remarkably well. Here, we present an advanced VS framework—automated hit identification and optimization tool (A-HIOT)—comprises chemical space-driven stacked ensemble for identification and protein space-driven deep learning architectures for optimization of an array of specific hits for fixed protein receptors. A-HIOT implements numerous open-source algorithms intending to integrate chemical and protein space leading to a high-quality prediction. The optimized hits are the selective molecules which we retrieve after extreme refinement implying chemical space and protein space modules of A-HIOT. Using CXC chemokine receptor 4, we demonstrated the superior performance of A-HIOT for hit molecule identification and optimization with tenfold cross-validation accuracies of 94.8% and 81.9%, respectively. In comparison with other machine learning algorithms, A-HIOT achieved higher accuracies of 96.2% for hit identification and 89.9% for hit optimization on independent benchmark datasets for CXCR4 and 86.8% for hit identification and 90.2% for hit optimization on independent test dataset for androgen receptor (AR), thus, shows its generalizability and robustness. In conclusion, advantageous features impeded in A-HIOT is making a reliable approach for bridging the long-standing gap between ligand-based and structure-based VS in finding the optimized hits for the desired receptor. The complete resource (framework) code is available at https://gitlab.com/neeraj-24/A-HIOT.

**Graphical Abstract:**

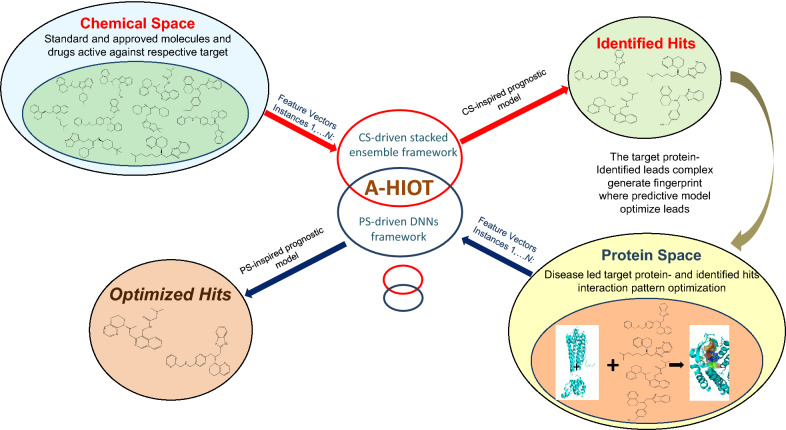

**Supplementary Information:**

The online version contains supplementary material available at 10.1186/s13321-022-00630-7.

## Introduction

Drug discovery refers to the invention or synthesis of new potential medications with pharmacological effects against pathological conditions. The entire process is expensive and challenging. Despite advances with impressive growth in technology, designing high-throughput screening experiments for all known compounds for a particular target(s) is not feasible[1, 2]. The identification of interactions between drugs and protein binding sites is crucial for developing new drugs[3]. It is to be noted that only a small fragment of 10^60^ molecules in the chemical space can therapeutically retain drug-like attributes, and it concludes the complexity of the drug discovery mechanism. Additionally, it is a lengthy mechanism, and the most elaborate task is lead molecule identification, which initiates the entire process[4, 5]. In earlier times, hit identification was predominated by high throughput screening (HTS), which was experimentally lengthy, time-intensive, and expensive. With the exponentially increasing and availability of protein structures and ligand resources, and computational architecture[6, 7], the HTS was conquered by virtual screening (VS)[8], which is a computational data-driven approach for the hit or lead identification. The VS is a computational algorithm-driven approach that curates drug-like compounds or molecules from ultra-large virtual libraries that can actively interact with the desired target, predominantly receptors or enzymes[9]. The VS is of two types: (a) ligand-based VS (LBVS), where the method relies on the similarity between molecules of interest and active molecule, and (b) structure-based VS (SBVS), where the selection of molecules depends on the interaction between molecules of interest and binding-site of desired protein structure. VS uses a combination of features based on the chemical, biological, and topological properties of selected molecules or targets as an input to model the interactions between the molecule and targets[10]. However, the drugs screened using VS techniques report falsely predicted molecules underperformed in the clinical trials, leading to resultant failure due to multiple reasons including varied pharmacokinetics and pharmacodynamics profiles may have a chance of failure in clinical trials [1, 11–13]. Therefore, the occurrence of ample false-positive (FP) and off-target hits is a significant limitation of previously discussed approaches[14]. Machine learning (ML)-infused artificial intelligence (AI) has been implemented in drug discovery. The integration of ML in VS has advanced the drug discovery discipline for more than two decades and assisted in diverse aspects, such as chemical and biological aspects, physical representation, drug repurposing, drug-target interactions, bioactivity, and binding affinity predictions[15–18]. ML handles structural or non-structural data resulting in precise interpretations. Deep neural networks (DNNs) have enhanced the AI domain that resulted in extensive applications in the field of drug discovery with commendable results while solving complex datasets (images or numeric), processing information, and providing inference abstraction[19]. The DL/DNN frameworks have been successfully applied in LBVS using classic statistical techniques and have reported superior performance[20]. However, DL algorithms also inherit adversities when implemented without manual parameter adjustments, which results in outcome redundancy; moreover, prognostic uncertainty remains a standing task that needs to overcome in the respective field. Hence, there is the demand for modern technological surge in the machine-intelligence (MI) frameworks for drug discovery with robust computational architecture, evolving statistical calculations, modern protein-structure calculation techniques[15], interpretability in predictive models, and ligand structure handling methods.

One such kind of advanced ML algorithm, the instance-based learning (IBL) that relies on the similarity and classification functions[21] where top-performing training instances are saved and used to predict a novel set of instances until the generalization set of time. Therefore, IBL may allow a set of rules for achieving higher accuracy among memory-based ML algorithms. However, IBL learns only from a group of stored instances and classifies, and as a result, it suffers from limitations regarding the application in drug discovery, with only one set of instances used at a time. Herein, we can try to strengthen the IBL in our study by incorporating advanced high-end computing and machine intelligence (MI) frameworks[22]. This kind of instance-based approach[23] has yet to be applied for drug discovery for enhancing transparency in the drug-target model that will reveals the presence/absence of crucial molecular features responsible for prediction results. Furthermore, a combination of primary ML and DNN frameworks, wherein individual frameworks are concatenated[24] and automated for synergized task execution that can enhance the real predictive power of the final model and be the future of AI. The combination of multiple prediction by any ML algorithm of frameworks called as ensemble, and, in general, the ensemble stands for togetherness. The performance of ensemble depends upon: (a) individual performance of base-learners, (b) diversity or independence of base-learner’s results of each other. The ensemble learning techniques includes; bagging, boosting and stacked generalization[25]. The ensemble learning has been diversely applied to drug discovery discipline and reported elsewhere[15, 26–30]. The ensemble algorithms used widely for QSAR model development, drug-target interaction predictions and protein–ligand binding pose[15, 31].

Several methodologies have been developed to handle and strengthen massive data for individual approaches that employ chemical and protein space to reduce false hits[10, 32–36]. The chemical space (CS) stands for an array of structurally significant molecules possessing relevant properties for a specific or set of biologically defined targets. The CS justifies multi-criteria objectives for ideal model development that can pave the way for hit identification from large VS libraries[16–18]. The ligand-based or CS-inspired hit identifications have been reported in DeepChem[37], AMPL[38], and, PyRMD[39]. The protein space (PS) is a collection of various features relative to ligand-binding modes, the binding pocket, and the type of protein–ligand interactions. The PS combined with ML, and /DNNs is also called modern SBVS[40, 41]. The PS acts as a filter for hit molecules optimization and is reported in various methods, including DeepVS[42], DeepDocking[43], and Deep Affinity[44].

**Fig. 1:**
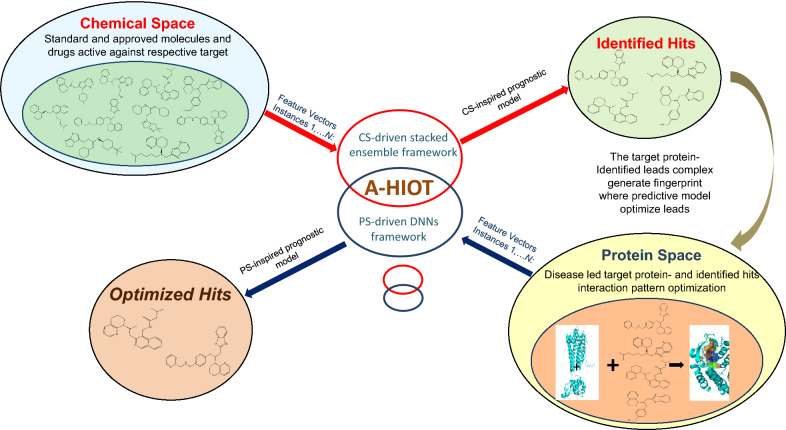
Graphical representation of the A-HIOT workflow and function. Automated-hit identification and optimization tool (A-HIOT) utilizes both ligand and receptor-structure information to bridge the long-standing gap between ligand-based and structure-based virtual screening. The input data for A-HIOT comprises marketed, FDA-profiled, and molecules under clinical trial for an individual or set of specific protein targets belonging to a similar family. The ligands were transformed into feature vector (*x*_*n*_ = 1,…, *N*) representation. The data preprocessing retains dimensionality and yields a machine-readable dataset. The machine and deep learning comprise the stacked ensemble framework in which random forest, extreme gradient boost serves as base-learners, and deep neural networks (deep learning) serves as the super learner job. The inhibitors-like representative feature-instances, hence represented, as chemical space (CS) module creation, result in the high-performance classification of the predictive model (CS-driven stacked ensemble framework). The true positive (TP) molecules are identified leads/hits that serve as input for the protein space (PS) module implemented in the A-HIOT framework. The identified leads were further explored for binding patterns implementing docking-simulation within the receptor pocket. The binary fingerprints for each protein–ligand complex are reckoned to assess the binding pattern. These fingerprints serve as deep neural networks input and outcomes a robust predictive model (PS-driven DNNs framework). The true positives obtained were further concatenated with protein–ligand interaction profiles and re-ranked as per the binding interaction (interaction number between protein–ligand complex) threshold. The collected molecules are optimized leads serves the purpose of final output in the A-HIOT framework

So far, previously discussed CS and PS methodologies have been developed separately or in hybrid manner on different platforms resulting in few satisfactory outcomes for identifying hit molecules and has been reported elsewhere[45]. However, we believe that integration of CS and PS leveraging IBL on a single platform for feature learning would identify and optimize hits simultaneously with higher accuracy and can be convenient for users easy to apply. On this trending hypothesis, we conceptualized a future-oriented VS framework—automated hit identification and optimization tool (A-HIOT) comprise of the stacked ensemble[46], deep learning architectures[19] and combines conventional approaches based on the chemical space (AI-driven predictive model derived from standard ligand information for respective targets) and protein space (target structure and interaction information collection constituting PS and AI-driven predictive model extracted from the interaction pattern of target protein–ligand complexes) (Fig. 1).

Following the proposed A-HIOT concept, the primary input requirement for A-HIOT are target selection and systematically profiled ligand collection. We chose CXC chemokine receptor 4 (CXCR4) as our drug target because its expression has been observed in multiple types of cancers, including breast, lung, and prostate cancers[47–51]; moreover, there are extensive discussions in the literature regarding active/inactive molecules against the CXCR4[52–54]. These prior observations led to significant interest in the development of CXCR4 inhibitors for developing this A-HIOT framework. We compiled the reported molecules active against CXCR4, along with the half-maximal inhibitory concentration (IC_50_). For generalization purpose, we have evaluated A-HIOT on diverse family of GPCR receptors. To overcome the lack of methods comparison in selecting optimized hits, we have also tried to assess the strength of A-HIOT with other ML/DL algorithms on protein receptors under study. Thus, the developed A-HIOT framework can be largely represented for classification and retrieve optimized hits/leads for any user choice of fixed target protein. The feature-based interpretability and classification process of the A-HIOT assists to overcome the black-box issue and followed the principles of explainable AI (XAI)[23]. In addition, our developed A-HIOT framework can be applicable for drug repositioning according to the current demand[55].

## Material and method

**Fig. 2:**
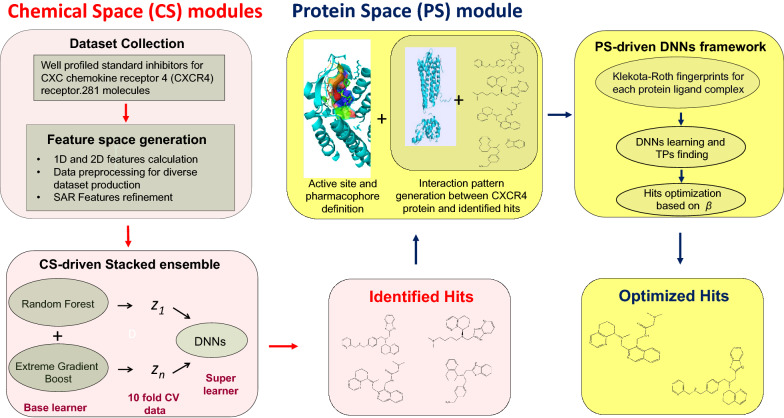
Methodology workflow. The workflow of chemical- and protein- space modules is shown as per connection and combination to obtain optimized hits/leads

Our rationale behind considering an individual chemical space that the molecules active against a specific receptor must inherit a particular structural feature pattern responsible for their biological action. The CS-driven stacked ensemble framework was established for enhanced classification performance for identification of hits/leads. The protein space comprises a well-defined protein target structure and identified hit/lead molecules active against the respective drug target. For a fixed target, active molecule selection requires refinement and analysis of various parameters, e.g., protein functional pocket, chemical nature of amino acids comprising the active site, prioritizing amino acid residues participating in drug binding at the target site, and the nature of interactions. Therefore, to integrate and sharpen the chemical-space performance, we used automated molecular docking, fingerprint-based feature vector extraction for amino-acid residues participating in ligand-target binding, followed by DNNs dependent optimized hit/lead molecule selection (Fig. 2).

### Training and evaluation dataset collection

As per proposed concept, the primary requirement is molecules already in the market, well profiled, and may be used for patient treatments. Therefore, we collected approved and well-known agonists of the proposed receptor CXCR4 along with the IC_50_ values from the literature. Molecules were collected and compiled, and three-dimensional (3D) structures were generated using openbabel (OB)[56], and energy optimization (*obminimize*, an OB module) was used with the steepest descent method for 500 steps using the Merck molecular force field, MMFF94s[57]. The molecules were stored in.mol2 format, and the overall dataset resulted in 175 molecules (Additional file 6: Table S1), which were referred to as the training dataset. The processed molecules were labeled as inhibitors (1 or positive) if IC_50_ < 0.05 µM, and the remaining were non-inhibitors (0 or negative), resulting in a dataset consisting of 81 inhibitors and 94 non-inhibitors (Additional file 6: Table S1). We prepared an independent evaluation dataset, retrieved from a directory of useful decoys-enhanced (DUD-E)[58], comprises 56 molecules, specifically for the CXCR4 receptor, classified as per their IC_50_ value out of which 43 were inhibitors (1/active) and 13 non-inhibitors (0/decoy). The evaluation dataset was prepared as per the training dataset (Table 1).

### Benchmark dataset

We prepared benchmark dataset and retrieved from DUD-E, which is a mixture of molecules actives and decoys against family of GPCR receptors, i.e., adenosine A_2A_ receptor (AA2AR) and CXCR4. We compiled these molecules together to evaluate the generality of A-HIOT framework. The dataset is comprising of 3415 molecules, classified as per their IC_50_ value consists of 115 inhibitors (1/active) and 3300 non-inhibitors (0/decoy). The benchmark dataset was prepared as per the training dataset.

### Data preprocessing and input generation for ML model

Molecular descriptors (1D, 2D) were calculated using PaDEL-Descriptor, an open-source tool[59]. In total, 1444 1D and 2D descriptors were extracted for each molecule in the dataset. The pre-processing steps were implemented for the initial dimensionality reduction to ensure data rigidity[60]. Descriptors with more than 85% zeros and descriptors with a standard deviation of less than 3% were removed. The PCC ($\rho$) was calculated in the R platform (https://www.R-project.org/), using the *corrplot* package, and the descriptors with $\rho$> 90% were considered redundant and removed. Data preprocessing produced a final dataset that was ready for ML modeling.

### CS-driven stacked ensemble architecture

The standard notation is used to define the data and classifiers:

*Ɗ* represents the training dataset comprising *N* (preprocessed feature vector dataset) known instances of input and response variables:

1 $${\mathcal{D}}= \left\{\left({x}_{n,}{y}_{n}\right),n=1,\ldots N\right\}, x\epsilon \phi , y$$

Here, *x* is the input consisting of *D* feature vectors (molecular descriptors and fingerprints),$\phi \text{is the feature space}$, *y* is the coupled response variable, and *x*_*n*_ represents the n-th feature vector of the instance.

The stacked ensemble architecture was established using the *H2O* library in *R* package (https://h2o-release.s3.amazonaws.com/h2o/rel-zipf/2/index.html)*.* The stacking framework collects uncorrelated predictions of base classifiers by strengthening diverse predictions and reducing overfitting in the final predicted model. While handling small datasets in biological and medicinal research, the crucial element quantifies stochastic and epistemic unpredictability. The ensemble architecture that we established constructs a consistent yet powerful method that can process the issue effortlessly. The approach is explained as follows using conventional notations:

*Base-learner data* (*tier*-0)*:* The training dataset, as represented in (Eq. (1)), is received as input for base-learning data. Considering cross-validation, the dataset ${\mathcal{D}}$ is further split into test set (${{\mathcal{D}}}^{j}$) and training set (${{\mathcal{D}}}^{-j}$), where ${{\mathcal{D}}}^{(j)}$⊂${\mathcal{D}}$ *j-*th fold of the dataset. The *J-fold* cross-validation dataset was drawn stochastically, splitting ${\mathcal{D}}$ into *J* approximately uniform fractions.

*Base learners* (*tier*-0 *learner, h*_*t*_): The base classifiers *h*_*t*_ comprise *T* base-learning algorithms, where *t* = 1,…,*T*, which learn from tier-0 data. The RF (*£*_*RF*_) [61] and XGB (*£*_*XGB*_)[62] algorithms were selected as the base learners for tier-0 learning procedure. The training occurred by invoking T algorithms on the training set (${{\mathcal{D}}}^{-j}$). The training output for *h*_*t*_^*(−j)*^ on *x*_*n*_ observations is *z*_*nt*_ and is concluded as follows:

2 $$z_{nt} = h_{t}^{( - j)} (x_{n} )$$

*Super-learner data* (*tier*-1*,* ${\mathcal{D}}$_*cv*_)*:* The input data for the super learner is emanated from ${\mathcal{D}}$ by leveraging the cross-validation results of the base learners. The cross-validation generated a new dataset for level-1 learning as:

3 $${\mathcal{D}}_{cv} = \left\{ {\left( {z_{n1} , \ldots ,z_{nT} , \, y_{n} } \right), \, n\, = \,1, \ldots ,N} \right\}$$

The vector dataset generated (*z*_1_,..., *z*_*T*_) for the base classifiers *h*_*t*_ was used as a meta-learner input.

*Super learner* (*tier*-1*, H*): This is also termed a *meta-learner* and is a weighted (*w*_*b*_) combination of base learners. For the given *x* vectors and the respective response variable y, *H* can be calculated as

4 $$H:y = {w_b}h\left( x \right) + \varepsilon$$

where *w*_*b*_ (*b* = 1,…,*B*) indicates the weights assigned to base learners, *h*(*x*) (*h*_*t*_(*x*)…*h*_*T*_(*x*)) indicates the base-learner vectors, and ε is the normal distribution error. The DNNs as a super learning algorithm (*£*_*DL*_) was chosen for ensemble study where the input data of H would be ${\mathcal{D}}$_*cv*_. The new instance (test set) prediction task was performed using *h*_*t*_ of the model, combined with *H*.

The entropy measure (E)[46] was used to assess the diversity of the ensemble framework. E varies in the range of 0–1 and is calculated as follows:

5 $$\mathrm{E}=\frac{1}{N}{\sum }_{i=1}^{N}\frac{1}{T-\left[T/2\right]}\mathrm{ min}\{{\theta }_{i}(T-{\theta }_{i})\}$$

where ${\theta }_{i}$ is the number of classifiers that misclassify the instance *x*_*i*_, *T* is the number of total classifiers, *N* is the number of samples. When *E* reached the value of 1, the abovementioned parameters were added to assess the classification performance of the ensemble in terms of the BCR, which is a modified version of the correct classification rate[33]; the BCR is considered as it dictates the highest diversity.

6 $${\text{BCR}} = \frac{{{S_e} + {S_p}}}{2}*(1 - |{S_e} - {S_p}|)$$

The sensitivity (*S*_*e*_) and specificity (*S*_*p*_) were considered while calculating the BCR. Higher BCR scores indicated the best-balanced classification model.

### Active site definition and binding mode sampling

To design a VS pipeline, the 3D protein crystal structure of CXCR4 retrieved from PDB[63] as PDB ID: 3ODU was used. Here, the critical issue was to explore and validate the active pockets and constituent amino acid residues of the protein. Therefore, we established a structure-based pharmacophore using Cavity V1.1 [64] and Pocket v3 [65], which are stand-alone tools. Further, an automated docking simulation was carried out to sample binding modes using AutoDock Vina [66]. Protein structure and pre-docking preparations were performed using the AutoDockTools [67] wizard. We selected first protein–ligand interaction complex from top 10 poses.

### Fingerprint calculation and DNNs architecture for optimized hits selection

The protein–ligand complexes were collected and compiled as complex datasets and binary fingerprints, the Klekota–Roth fingerprint count[68], the substructure count for each complex was calculated using the PaDEL-Descriptor software. The complex dataset was further used to predict interactions (*d*_*i*_) between target proteins and molecules using the stand-alone tool, protein–ligand interaction profiler (PLIP)[69]. The instance-based DNNs algorithm was implemented in R environment and employed using the *H2O* library. The training dataset is as follows:

7 $$D= \left\{\left({x}_{n,}{y}_{n}\right),n=1,...N\right\}, x\epsilon \phi , y$$

The algorithm was first trained for 50 epochs with three hidden layers having consecutive (400, 200, 400, and 2) neurons, each using the *“Tanh”* activation function for the first three layers, followed by fivefold cross-validation. The grid search-based hyperparameters optimization for high predictive accuracy, classification performance, and best model selection with refined parameters was further applied. The advanced parameters, such as *momentum training, rate annealing, and regularization* (*input dropout ratio*), were separately defined using the hyperparameters. The training dataset (*D*) was input into the input layer *α*, and weights (*w*_*i*_) and bias (*b*) were assigned to each information and bias:

8 $$\alpha ={\sum }_{n=1}^{n}{\mathrm{w}}_{\mathrm{i}}{x}_{i}+ b$$

To obtain the classification output, *f*($\alpha$) and PLIP interaction numbers *d*_*i*_*,* where *d* = *d*_*1*_,…*d*_*i*_*,* were concatenated as follows:

9 $$\beta = f(\alpha )+( {d_{i} })$$

As per the structure-based pharmacophore and crucial amino-acid residue participating in (CXCR4 and IT1t complex, standard ligand bound in PDB file) interaction, a threshold value for (*di*) was decided; each complex was assigned a *d*_*i*_ value. The final curation for best-performing molecules were concluded, where β is the summed selection score.

### Training, model validation and benchmark study

The CS-driven stacked ensemble architecture was trained with feature vector dataset represented in Eq. 1. Firstly, the dataset used by base learners to produce cross-validated output as described in chemical space module section. Secondly, the cross-validated dataset used as input by stacked ensemble, where a three-layered DNN (200, 400, 2) was used as the super learner, and the "*Tanh*” activation function was employed for the first two layers for 50 epochs. The number of accurately classified or true positives (TPs) molecules were identified hits. The molecules in the TPs and TNs classes of the classification process were extracted and used as input for the next step, which was PS-driven DNNs framework for hit/lead optimization employing protein–ligand interaction scores (β). The molecule dataset used for docking simulation with the CXCR4 protein structure to explore protein–ligand binding patterns and collect their complex structures. The number of interactions (*d*_*i*_) protein–ligand binding complexes were collected using PLIP. The Klekota–Roth fingerprint count was also calculated, and the fingerprint dataset was used to train advanced four-layered DNNs. The molecules classified as TPs were further merged with *d*_*i*_, and active molecules were finally selected according to the value of β. The protein–ligand interactions and molecules were visualized using PyMoL. The framework evaluation and benchmark experiment method details are given in Additional file 1.

### Evaluation metrics

The classification of the developed framework was assessed using the different performance measures viz., accuracy, specificity, sensitivity and area under the curve (AUC) of receiver operating curve (ROC) for the CS as well as PS modules. The number of accurately classified or true positives (TPs) molecules in chemical space module were identified hits. The TPs of proteins space module bound with *d*_*i*_*,* were selected as optimized hit/lead molecules.

### Comparison with other ML algorithms

The performance of CS-driven stacked ensemble framework for hits/leads identification was compared with other ML classification algorithms comprising RF, XGB and DNNs. The comparison task was implanted in R platform. The technical details are given in Additional file 1. The performance of PS-driven DNNs framework for hits/leads optimization was compared with other ML classification algorithms comprising RF and XGB.

### Independent case study

To test and demonstrate optimized hits selection power of A-HIOT we considered androgen receptors (AR). We compiled well-profiled molecules active against AR along with IC_50_ from accessing AR binding dataset (https://www.fda.gov/science-research/endocrine-disruptor-knowledge-base/accessing-ar-binding-dataset-androgen-receptor) and NRLiSt [70] database for training purpose. The training dataset comprise of 146 active (1) and 157 inactive (0) and in sun 303 molecules. To evaluate the capability of A-HIOT for eliminating decoys and false hits as well as selection of strong optimized hit, an independent dataset was compiled and retrieved from DUD-E database. The test dataset comprises of 249 active and 872 inactive and in sum 1122 molecules. The training and testing dataset were pre-processed as per previously disclosed concept. The pre-processed molecules generated ML-ready dataset for CS-module of A-HIOT. We retrieved 3D protein crystal structure of AR as PDB ID: 2AM9 from PDB database for PS-module A-HIOT.

## Results and discussion

Integrating chemical- and protein-space-driven architectures can simultaneously lead to the identification (by CS module) and optimization (by PS module) of hit molecules, achieving the A-HIOT framework, which stands for automated-hit identification and optimization tool. The A-HIOT uses multiple R libraries to develop stacked-ensemble and DNNs algorithms.

The A-HIOT implements CS-driven stacked ensemble framework (CS module) comprising RF and XGB as base-learners and DNNs as super-learner, where the weight of every base model has deemed a random variable for chemical space. The ensemble algorithm within the A-HIOT does not inherit probabilistic nature, which allows us to effectively explore the integration of R libraries to obtain the best accuracy and specificity of the predictive model. It is to be noted that features should be diverse so that their (features) information would not hinder the capability of the predictive model. Feature engineering is a prime requirement of CS module for achieving satisfactory performance, interpretability of the predictive model, and overcoming dimensionality[60]. The molecular features inherits calculated quantitative values of molecular structures that perhaps correlate to the biological activity of the respective structure; one-dimensional (1D) and two-dimensional (2D) features can be easily calculated and are interpretable and understandable[71, 72]. The initial feature space consists of 63 classes (Additional file 7: Table S2) that was further preprocessed as per methods section to generate ML-ready dataset is engineered, along with rigid dimensionality. The final input dataset comprises of 674 features related to 38 classes.

The docking simulation establishes interaction patterns among target protein and identified hit/lead molecules; interaction-dependent fingerprints allow us to assemble the PS-driven DNNs framework. The DNNs produces a predictive model that can effectively classifies molecules by adjoining with interaction numbers**,** re-ranked and the best-performing molecules were picked-up as optimized hit/lead molecules. The weighted ensemble and interaction fingerprint-dependent DNNs predictive framework produce a simple, hitherto strong, in silico pipeline to eliminate uncertainty while achieving lead identification and better selection during lead optimization. We have then assessed CS and PS modules of the A-HIOT in the corresponding section and comparison was carried out.

### Performance of CS module of A-HIOT and comparison

**Fig. 3:**
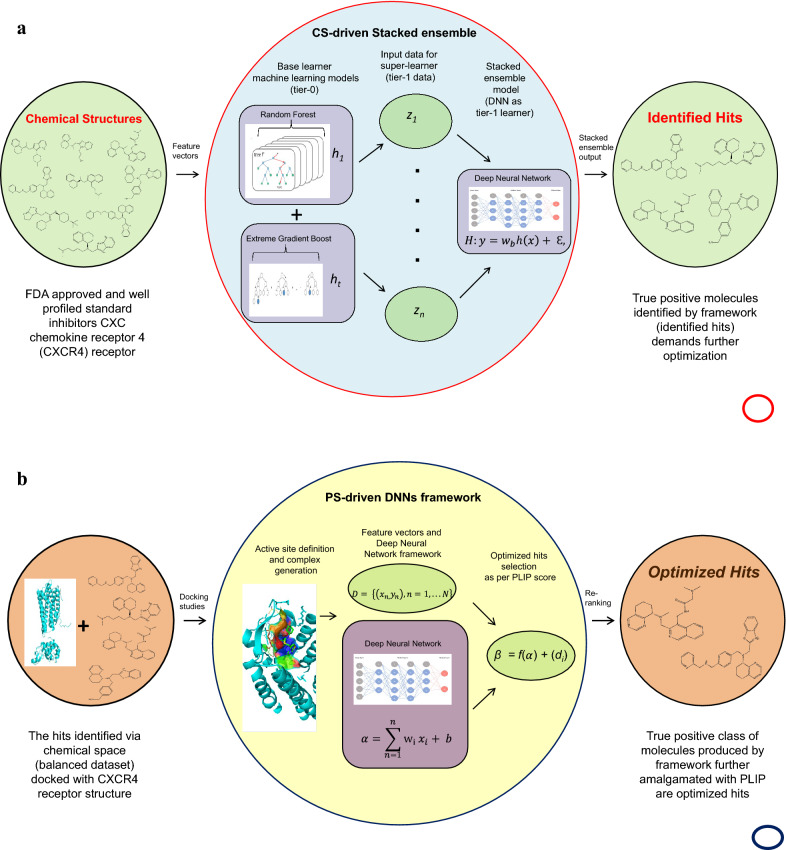
**a **Chemical space module architecture for hit/lead identification. The first module of A-HIOT identifies hit/lead molecules emphasizing chemical space (CS). Here, as per concept, the chemical structures of known inhibitors for CXCR4 protein were collected, transformed into feature vectors, and preprocessed to achieve a machine-readable dataset. The chemical space leverages random forest (RF), extreme gradient boost (XGB), and deep neural networks or deep learning (DNN/DL) algorithms to construct a predictive classification model. We combined these distinctive models into the stacked ensemble where RF and XGB serve as tier-0 learners, receive input data as feature vectors, train *h*_*1*_*… h*_*t*_ predictive models and produce *z*_*1*_*…z*_*t*_ predictions. The tier-0 predictions serve as input for the tier-1 learner that is DNN (*H*). The tier-1 algorithm is termed a meta-learner. The *w*_*b*_ (*b* = 1,…,*B*) indicates the weights assigned to base learners, *h*(*x*) (*h*_*t*_(*x*)…*h*_*T*_(*x*)) indicates the base-learner vectors, and *ε* is the normal distribution error. The true positives produced by the CS-driven stacked ensemble framework were the identified leads/hits because the framework learned the inhibitors-like representative feature instances that resulted in a high-performance classification prognostic model. This step ensures reducing the huge and complex dataset to a meaningful one that still demands further optimization. Thus, the CS-driven stacked ensemble framework in the A-HIOT framework achieves hit identification and is herein represented as the red ring. **b **Protein space module workflow for hits/leads optimization. The second protein space (PS) module of the A-HIOT optimizes hit/lead molecules emphasizing protein–ligand interaction patterns. Initially, the protein structure is obtained and explored for potential binding sites, binding residues within the binding pocket. Furthermore, the balanced dataset collected from chemical space comprising true positives and true negatives. The interaction patterns are established among protein and identified molecules employing docking simulation. The binary fingerprints for each protein–ligand complex are reckoned to assess binding-pattern. These fingerprints serve as deep neural network input and a robust predictive model (PS-driven DNNs framework). The true positives produced by the model were further concatenated along with protein–ligand interaction profile (PLIP) score (*d*_*i*_) and re-ranked following binding interaction threshold. The collected molecules implemented in the A-HIOT framework named optimized leads are represented as the blue ring. We have devised this module using CXCR4 as a protein case under study. The $D$ represents DNN ready dataset where the DNNs output *f*($\alpha$) for the classification model. Further concatenation with (*d*_*i*_) yielded *β* that produced optimized hit molecules

We established a stacked generalization[73] ensemble and constituted the hit identification CS module (Fig. 3a) for the A-HIOT framework. The stacked ensemble speculates the weighted average of each consistent model of the ensemble framework, and a super learner tunes the weights over the feature space $\phi$ while integrating these models. The performance of CS-driven stacked ensemble framework was carried out in such a way, firstly, tenfold cross-validation was implemented to validate the dataset and evaluate the prediction efficiency. A random subset of 10% of the training dataset was selected, named the internal evaluation dataset; the rest was implemented for model training. The CS-driven stacked ensemble model was trained and internally evaluated, including accuracy, sensitivity, specificity, and AUC-ROC matrices.

The feature vector dataset (Eq. 1) was first used as input data. The base-learner frameworks then performed tenfold cross-validation (CV) and the CV output data was further served as input data for the super learner (*tier*-1*, H*) framework. The stacked ensemble achieved an accuracy of 0.948 for internal evaluation (internal test set), along with 0.961 sensitivity, 0.988 specificity, and 98.8% AUC.

We compared CS-module of A-HIOT with three diverse individual classification algorithms namely RF, XGB, and DNNs/DL. Firstly, the RF model obtained 0.826 accuracy, 0.891 specificity and 89.1% AUC for training performance (Additional file 2: Fig. S1, Additional file 8: Table S3) for internal evaluation (test set). Secondly, we used the XGB, for internal evaluation, the XGB framework classifies with an accuracy of 0.809, and specificity was found to be 0.761, and 81.2% AUC respectively, and shown in (Additional file 3: Fig. S2, Additional file 9: Table S4) Thirdly, we used DNNs employing grid-based hyperparameters tuning to dig deep into the respective algorithms for the best classification outcomes. The best model established an accuracy of 0.902, a maximum sensitivity of 0.896, specificity of 0.923, and AUC-ROC for internal evaluation was 91.4%, AUC respectively, for the internal evaluation dataset and shown in (Additional file 4: Fig. S3, Additional file 10: Table S5). Overall, it can be concluded with tenfold cross validation datasets, CS-module of A-HIOT performed much better than individual ML/DNN in terms of higher accuracy, specificity and AUC.

**Fig. 4:**
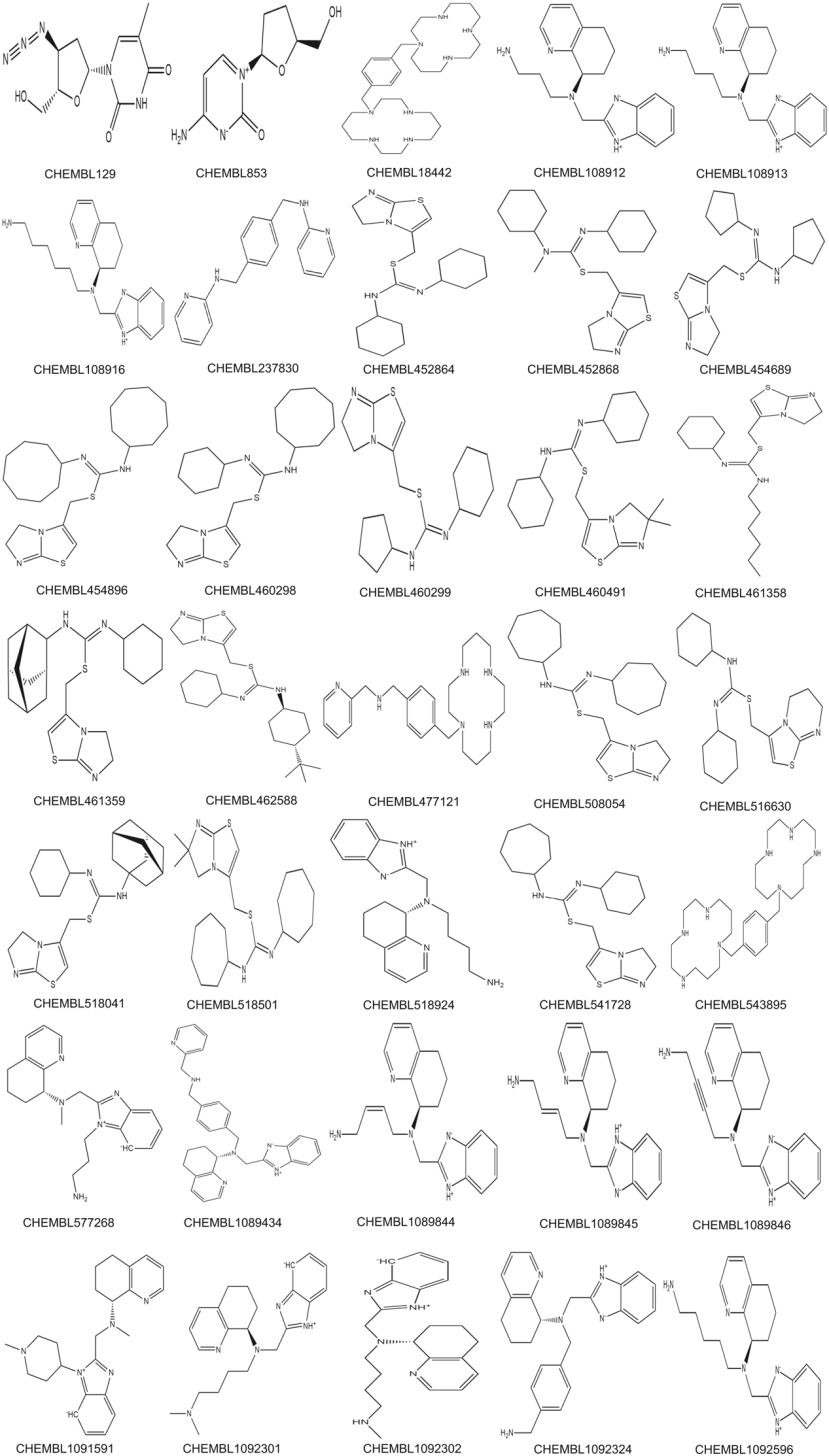
Identified hits by the CS-driven stacked ensemble framework from small independent validation dataset. The stacked ensemble of chemical-space A-HIOT led to the best identification of 35 hit molecules for a particular target (in case, CXCR4 receptor). The trained CS module of A-HIOT tested on small independent validation datasets (56 molecules) predicted most of the hits belonging to the aromatic ring system, in particular, imidazole ring which is the prime inhibitor of CXCR4 receptor justified its good performance measures as compared to individual machine learning algorithms

We have also assessed each framework on the small independent validation dataset, rigorously to determine the classification performance, feature learning, and hits/leads identification. The RF reported minimal overfitting as it obtained 0.726 accuracy and 0.747 specificity rate, XGB performed well by bringing 0.789 accuracy and 0.816 specificity, DNN disappointed by receiving merely 0.782 specificity rate and nominal over-fitting. As compared to individual frameworks, the CS-stacked ensemble module framework obtained 0.867 accuracy and 0.967 specificity on the small independent validation dataset and identified 35 hit/lead molecules (Fig. 4), showcasing comparative performance in Table 3. The stacked ensemble was found to enhance the classification performance in comparison to the individual framework.

**Fig. 5:**
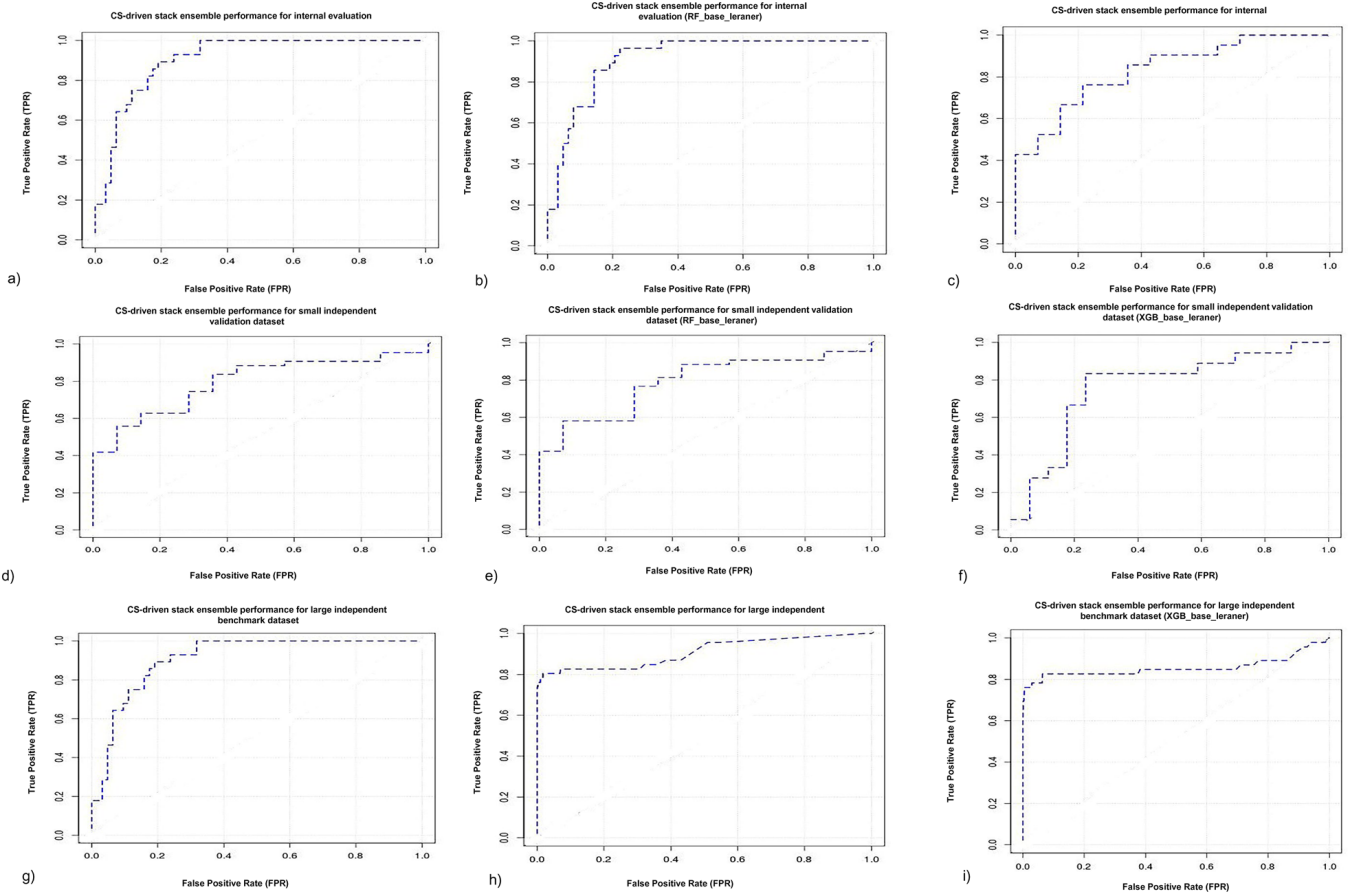
The CS-driven stacked ensemble framework classification performance. The stacking framework collects uncorrelated predictions of base classifiers, strengthening diverse predictions and reduce overfitting in the final predicted model. The results of internal testing and independent validation of the prognostic model were assessed by area under the curve- receiver operating characteristics (AUC-ROC). Herein, the AUC-ROC plots illustrate the augmented classification performance achieved by stacking framework instead of implementing a specific classification algorithm. For internal evaluation, the designated super-learner (DNNs) has obtained 98.8% AUC-ROC (**a**) while base-learners RF and XGB achieved 88.6% (**b**) and 79.6% (**c**) AUC-ROC, respectively. The trained and tested prognostic model administered to identify hits from a small independent validation dataset has achieved a remarkable 83.90% AUC-ROC for the stacked framework (**d**). In contrast, base-learners, RF, and XGB obtained 81.80% (**e**) and 80.82% (**f**) AUC-ROC. The benchmark performance AUC-ROC plots for the CS-driven stacked ensemble obtained 90.2% (**g**) and base-learners RF and XGB obtained 82.2% (**h**) and 81.3% (**i**). Our results from implementing different machine and deep learning algorithms suggested that if any of the algorithm cannot handle input data well, the super-learner could handle the classification and data tasks. From the independent dataset, CS module of A-HIOT identified 35 hit molecules those demand further optimization as per receptor structure and will be considered as input for PS module of A-HIOT

The reason for superior performance of CS-stacked ensemble module of A-HIOT in comparison with other individual ML/DNNs algorithms is picking up suitable hits for a particular target (35, Fig. 4); which could be likely potent inhibitors of CXCR4. The 3D-QSAR studies of CXCR4 receptor (PBD ID:3OE6, 3ODU) is well known and vastly employed in literature. The bound ligand found in PDB structure and structure–activity relationship (SAR) studies shows that the critical structural constituents, the prime requisites to be a desired ligand for CXCR4 comprises of: a) imidazole, imidothizoles or benzimidathiazole ring systems which helps in interaction with D97, E288, and D193 amino acid residues of CXCR4 binding site; b) a protonated nitrogen moiety helps in interaction with D97 and E288; c) one or more aliphatic moiety which would be six-, seven- or eight-membered ring system for optimal binding with W90, H113, and Y116 binding site residues. Essential or optimal aromatic ring system could be considered as: (i) quinazoline, (ii) purine, (iii) naphthalene, and (iv) indolyl[74]. The observations on small independent validation datasets revealed the correctly predicted hits belonging to the aromatic ring system in particular imidazole which is the prime requirement to be an inhibitor for CXCR4 that justified overall good performance measures of CS-module of A-HIOT as compared to individual ML/DNN algorithms (Fig. 5). The CS-driven stacked ensemble framework attained a significant, balanced classification rate (BCR) of 0.8. We collected TP and TN molecules to create a balanced dataset for the next step, i.e., the protein space module.

### Protein cavity and interaction-pattern analysis for PS

**Fig. 6:**
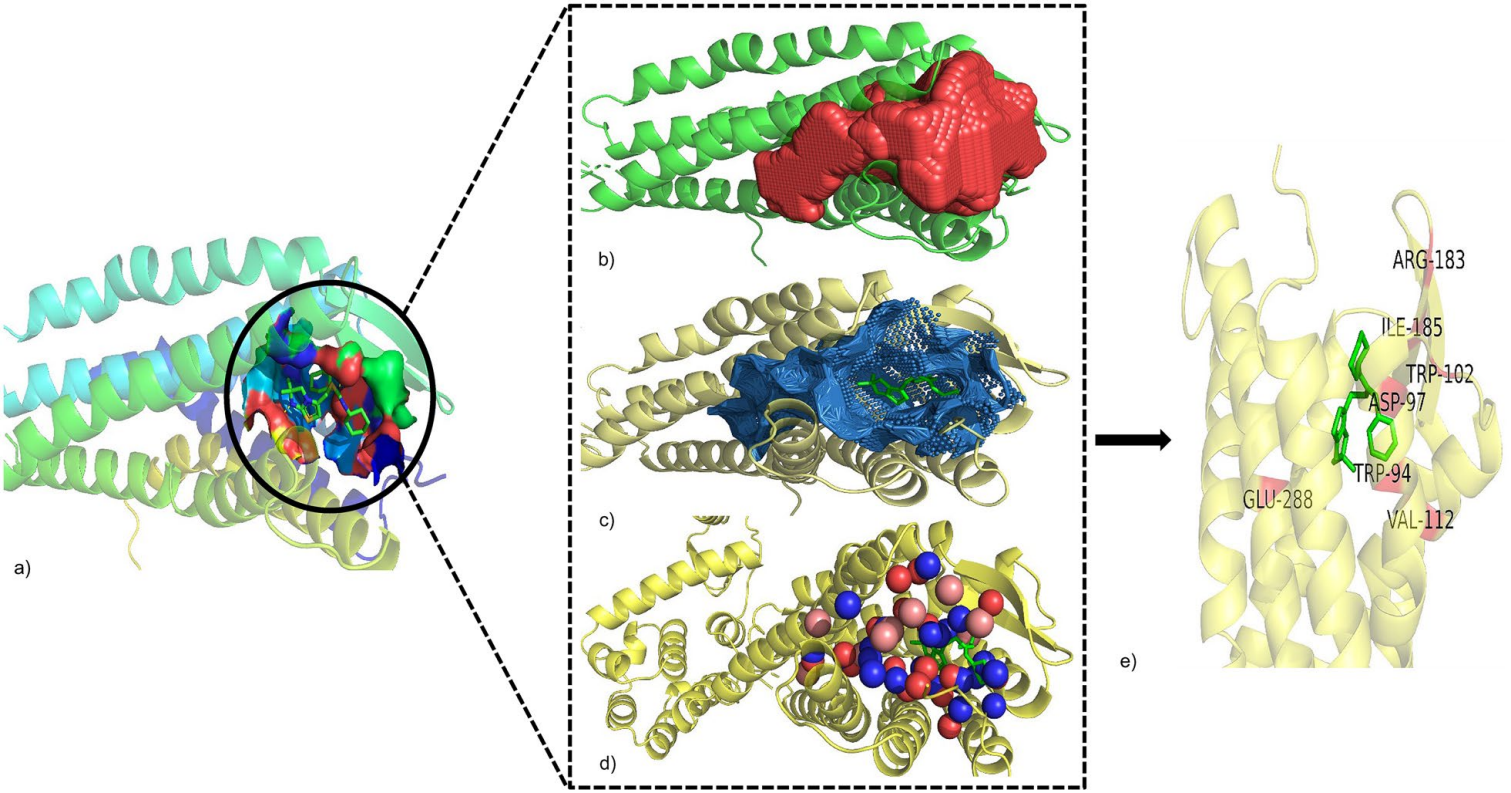
Binding pocket investigation and quantification for the target protein. Binding pocket assessment and crucial amino acid residues designation is vital for pharmacological activity by liable molecules. Herein, we displayed the binding pocket investigation and quantification layout. We first retrieved the 3D structure of CXCR4 (**a**) protein from the PDB database and bound ligand (PDB ID: 3ODU). The CXCR4 structure was subjected to the Cavity program to assess vacant ligand-binding pocket (red) and grid points (violet) as represented in **b** and **c**. For binding residue quantification, the results of the Cavity program were used as input by Pocket v3 that result in the amino acid residues location (**d**) along with probable types of interaction. The blue colored spheres symbolize hydrogen bond donor, red are hydrogen bond acceptor, and pink represents hydrophobic interaction residues. In summary, the active residues comprise the active binding pocket for CXCR4 protein (**e**) along with residue location. The information generated will serve as evaluation for assessment of PS-driven DNNs framework of A-HIOT with other machine learning algorithms

Before assessment of PS module (Fig. 3b) of A-HIOT, we want to clarify the usage of a target i.e. CXCR4 in detail. We have chosen CXCR4 receptor protein which itself is an essential regulator of immune system espionage and inflammation homeostasis, and its structure has been investigated along with the bound antagonist IT1t and cyclic peptide CVX15[75–77]. We retrieved the protein's crystal structure in the bound state with antagonist IT1t and carried out pharmacophore mapping, also called geometrical measurements of the active pocket. The pharmacophore features, including hydrogen bond donors, hydrogen bond acceptors, and hydrophobic sites, were collected and integrated with an active pocket definition (Fig. 6a–d). The active pocket comprises the following: (a) the critical amino acid residues encompassing the active pocket were W94, D97, W102, V112, Y116, R183, I185, C186, D187, and E288 belonging to the 7tmA_CXCR4 domain of the CXCR4 family; (b) other amino acid residues contributing to ligand binding were C28, Y45 (belonging to the CXCR4 N-terminus domain), V96, F93, Y121, R188, F248, Y256, I286, and F292 cross-referred via conserved-domain search (CD-Search)[78]. The functional pocket assessment determines the probable number of interactions among proteins and drug/ligand molecules and justified by interaction pattern analysis between CXCR4-IT1t complex, the standard ligand found in PDB structure, and a threshold value was proposed for the number of interactions; as per our hypothesis, the number of interactions per protein–ligand complex was 9–12 (Fig. 6e).

Additionally, docking simulation experiment generated diverse interaction patterns along with 10 poses and top pose with best interactions were first selected. The interaction profiling for the complex dataset was carried out by implementing PLIP, which designated the interaction accompanying the type of interaction to each ligand and complex. The interaction profile explains that W94, D97, W102, R183, R188, F248, Y256, E288, and F292 are critical amino acid residues that perform essential roles for the protein function. W94 have pi-stacking as well as hydrophobic interactions; D97 forms hydrogen bonds, hydrophobic interactions, and salt bridges; E288 forms hydrophobic interactions, hydrogen bonds, and salt bridges; R188 forms hydrogen bonds and pi-cation interactions; moreover, W102 forms hydrophobic interactions. The information generated will serve as evaluation for assessment of PS-driven DNNs framework of A-HIOT with other machine learning algorithms.

### Performance of PS module of A-HIOT and comparison

The calculated Klekota–Roth fingerprint count (4860) for the protein–ligand complexes dataset that computes imperative fragments or substructures for given dataset with refined biological vitalities and prepares as input for PS-driven DNN framework. The fingerprint approach was implemented for interaction rescoring, boosting the predictive power of the DNNs framework. The training using fingerprint data comprises of imperative substructure-encoded biological activity information[79]. The DNNs framework learns crucial features employing IBL and established the trained predictive model, further using internal test set, the classification evaluation (*x'*) was carried out and the predictive model obtained 0.819 accuracy, 81.2% AUC-ROC, 0.913 sensitivity, and 0.824 specificity during classification process. The TPs classified molecule in *x'* were adjoined with the PLIP score to achieve a sensible array of molecules, and further re-ranked as per defined pharmacophore descriptor threshold (9–12).

**Fig. 7:**
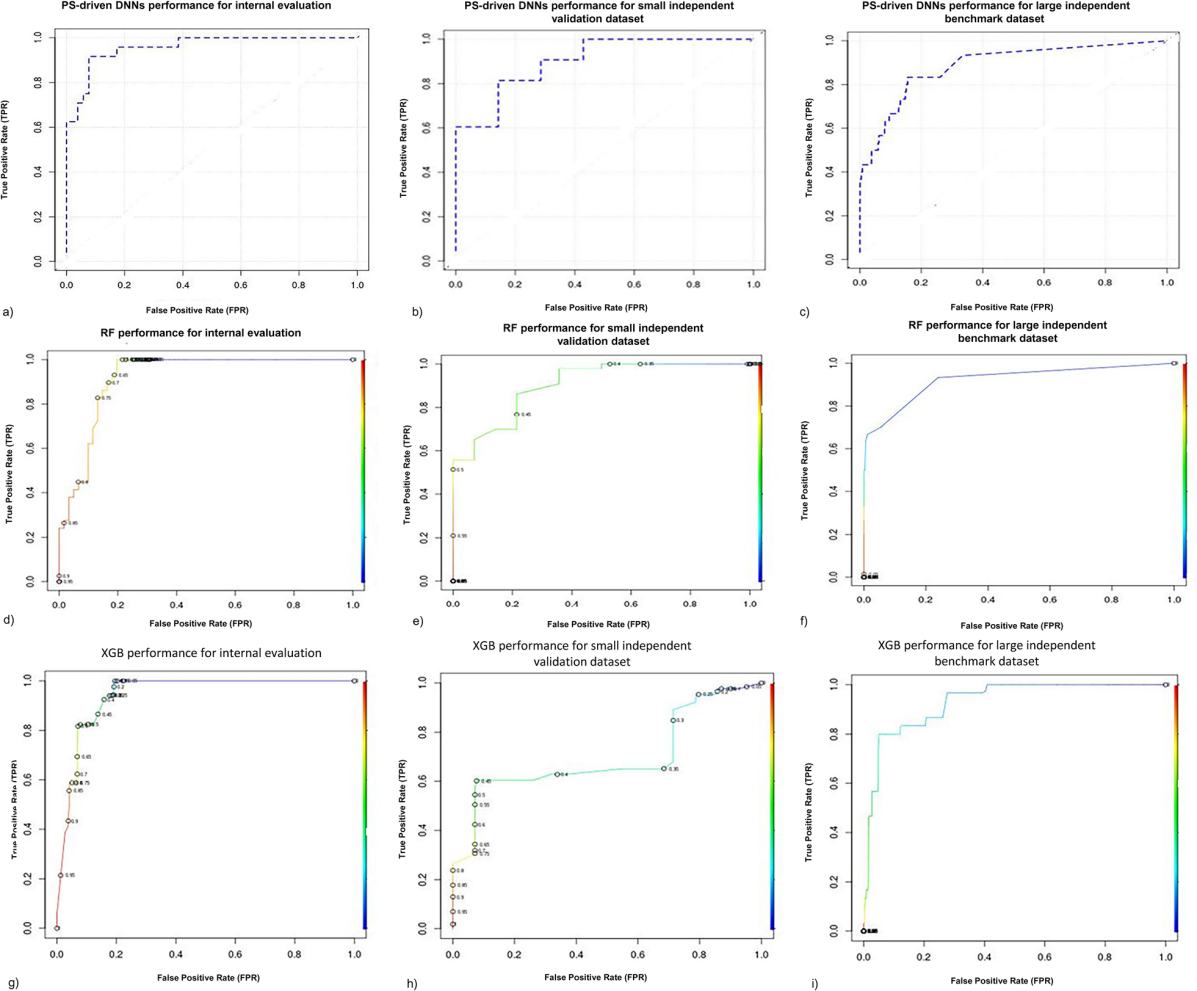
The PS-driven DNNs framework performance comparison. The PS-driven DNNs framework trained with binary fingerprint dataset encodes protein–ligand interaction pattern information, and prognostic model classify unlabeled dataset following interaction pattern information. The AUC-ROC plots shows the classification performance obtained by PS-driven DNNs framework for binary fingerprint dataset for internal evaluation 81.2% (**a**), for the small independent validation dataset 88.4% (**b**) and for benchmark dataset 89.8% (**c**)

**Fig. 8:**
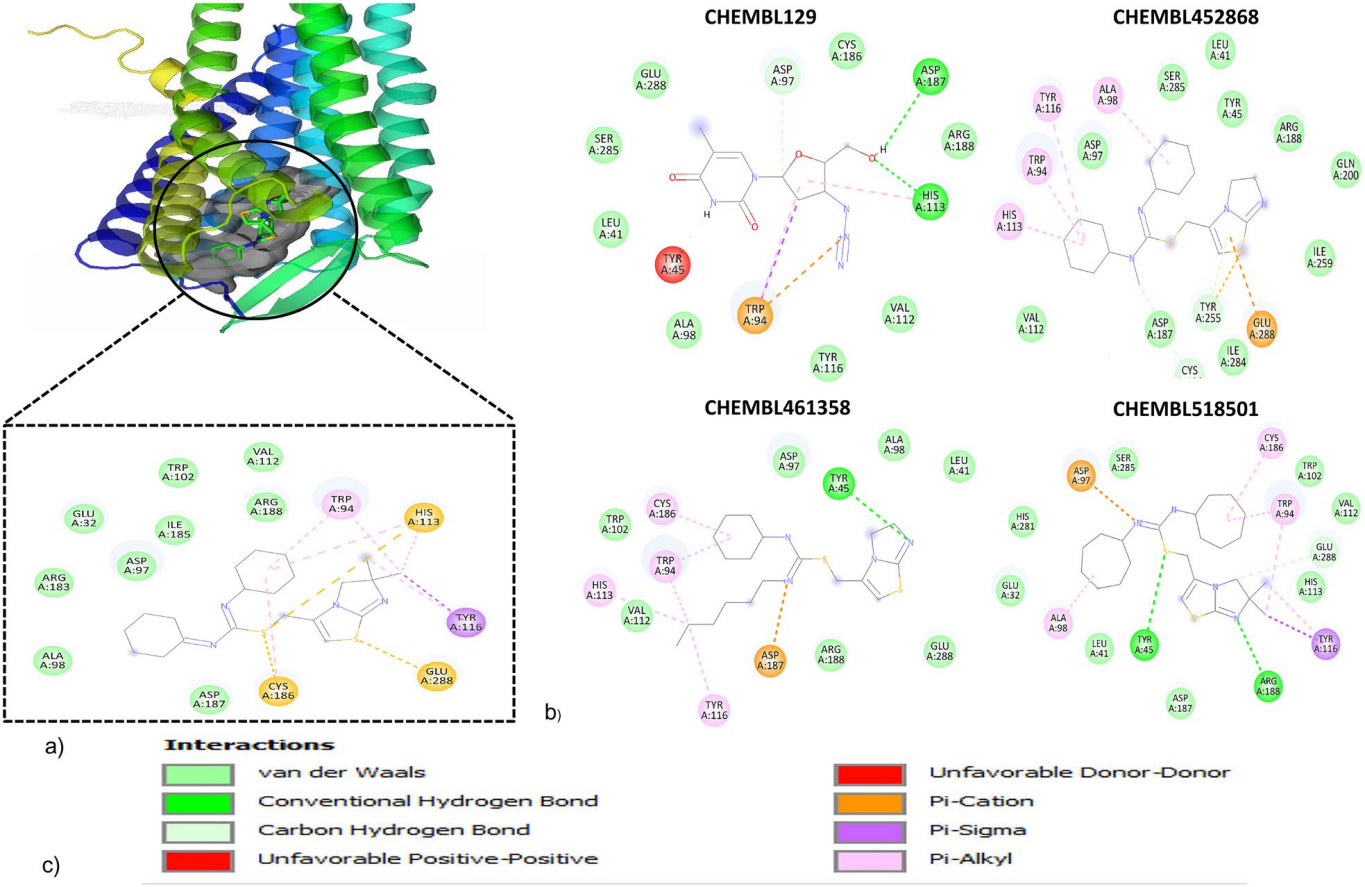
The representation of optimized hits by PS module. The true positives (TP) identified from PS-driven module of A-HIOT were decoded into molecule IDs, merged with PLIP score (di), and ranked, observing the proposed threshold for the CXCR4 binding interaction profile. The *β* illustrates the final ranking score for each ligand molecule subjected for optimization; **a** shows the CXCR4 interaction patterns and participating amino acid residues with its standard ligand (IT1t); **b** accumulates all four molecules (CHEMBL129, CHEMBL452868, CHEMBL461358, and CHEMBL518501) from independent set administered for optimization coupling interaction patterns and **c** details the types of interaction and bond formation pattern

We also carried out the PS-driven DNNs framework validation by utilizing the small independent validation dataset compared in Table 2. For validation dataset it obtained 0.859 accuracy, 88.4% AUC-ROC (Fig. 7a–c), and 0.872 sensitivity, and 0.822 specificity, where sensitivity denotes true positives (TPs) rate and TPs were the optimized hits along with bound IT1t standard ligand in PDB file (Fig. 8a–c). We found four best performing optimized hits from a small independent validation dataset.

**Table 1:** Summary of the molecular datasets used in this study

Dataset Name	No. of molecules	No. of active molecules (1)	No. of inactive molecules (0)
Protein class: CXC-chemokine receptor 4 (CXCR4)			
Training dataset*	175	81	94
Small independent validation dataset	56	43	13
Large independent benchmark dataset	3415	115	3300
Protein class: Androgen receptor (AR)			
Training dataset*	303	146	157
Independent test dataset	1121	249	872

^*^The training dataset partitioned into 7:3 classified as Internal test set (*x'*) for both CS- and PS-modules

**Table 2:** PS-driven DNNs classification performance: the comparison of classification performance of the PS-driven DNNs/DL framework for hit/lead optimization employing PS module

Algorithm	Dataset	Accuracy	Sensitivity	Specificity	AUC-ROC
PS-driven Deep Neural Networks (DNNs/DL)	Internal evaluation (*x'*)^a^	0.818	0.913	0.824	0.812
	Small independent validation dataset^b^	0.859	0.872	0.822	0.884

^a^Total of 175 (81 inhibitors and 94 non-inhibitors) partitioned into 7:3 classified as Internal test set (*x'*)

^b^46 (35 inhibitors and 11 non-inhibitors) classified as small independent validation dataset

For lack of availability of methods, the classification performance of PS-driven DNNs framework was compared with individual framework viz., RF and XGB algorithms for optimized hits/leads selection. The binary fingerprint feature vectors served the input dataset as Eq. 7. Firstly, RF algorithm used for internal evaluation (internal test set, *x'*) wherein, it obtained 0.802 accuracy, 0.754 sensitivity, 0.821 specificity and 80.1% AUC. Secondly, XGB used for internal evaluation where it obtained 0.806 accuracy, 0.786 sensitivity, 0.813 specificity and 81.2 AUC. The RF showed minimal specificity (0.488) and certain overfitting as it obtained large AUC (82.2%) along with 0.614 accuracy and 0.724 sensitivity for classification task. Similarly, XGB obtained small amount of specificity (0.534), low accuracy (0.631), insufficient AUC (69.9%) along with good sensitivity 0.763. The top ranked molecules identified by PS module of A-HIOT were optimized hits/leads according to the proposed pharmacophore mapping which unveils the interaction as well as interacting substructure counts. Moreover, the PS module is the amalgamation of the established protein cavity and protein–ligand interaction pattern profiles which were boosted by the DNNs framework in PS module of A-HIOT framework for hits/leads optimization justified its superior performance as compared to individual MLs (RF/XGB) (Fig. 7d–i; Table 4).

### Performance of A-HIOT for hit/lead identification and optimization on benchmark dataset

To ensure the generalization capability of the A-HIOT, we carried out performance analysis for CS and PS module framework for hits/leads identification and optimization from benchmark dataset that comprises of mixture of active molecules and decoys against family of GPCR receptors as mentioned in the materials and methods section.

The CS module achieved an AUC of 90.2% (Fig. 5) and accomplished the classification task remarkably, maintaining the balance between sensitivity (0.921) and specificity (0.987); additionally, the accuracy (0.962) of the entire model, shown in Table 3. The satisfactory sensitivity (measure of identifying hits/leads (TPs)) and specificity (measure of eliminating false hits/leads (TNs)) translates the predictive power of CS-driven stacked ensemble framework. We compared the benchmark performance among individual ML classification algorithms, shown in Table 3 and observed the higher accuracy of CS-driven stacked ensemble framework over the three individual frameworks as similar performance case of the independent small dataset.

**Table 3:** CS-driven Stacked Ensemble framework and Benchmark performance comparison for CS module: the performance comparison of CS-driven Stacked Ensemble framework and Benchmark with individual ML algorithm

Algorithm	Dataset	Accuracy	Sensitivity	Specificity	AUC-ROC
Random Forest (RF)	Internal test set (*x'*)^a^	0.826	0.793	0.891	0.891
	Small independent validation dataset^b^	0.726	0.642	0.747	0.807
	Large independent benchmark dataset^c^	0.914	0.705	0.823	0.823
Extreme Gradient Boost (XGB)	Internal test set (*x'*)^a^	0.809	0.819	0.761	0.812
	Small independent validation dataset^b^	0.789	0.571	0.816	0.782
	Large independent benchmark dataset^c^	0.908	0.827	0.709	0.787
Deep Neural Networks (DNNs/DL)	Internal test set (*x'*)^a^	0.902	0.896	0.923	0.914
	Small independent validation dataset^b^	0.894	0.877	0.782	0.866
	Large independent benchmark dataset^c^	0.924	0.767	0.923	0.951
Stacked Ensemble	Internal test set (*x'*)^a^	0.948	0.961	0.988	0.991
	Small independent validation dataset^b^	0.867	0.911	0.967	0.839
	Large independent benchmark dataset^c^	0.962	0.921	0.987	0.902

^a^Total of 175 (81 inhibitors and 94 non-inhibitors) partitioned into 7:3 classified as Internal test set (*x'*)

^b^56 (43 inhibitors and 13 non-inhibitors) classified as small independent validation dataset

^c^3415 (115 inhibitors and 3300 decoys (termed as non-inhibitors)) classified as large independent benchmark dataset

The PS module obtained an AUC of 89.8% (Fig. 7) and accuracy of 0.899 along with sensitivity and specificity of 0.902 and 0.924 (Table 4), respectively, demonstrating the effectiveness of using the interaction fingerprint as well as the accuracy of the predictive model in optimizing identified hits/leads. The interaction profile concatenation helped us to screen optimized hit/lead molecules. Thus, the advantages of utilizing both CS- and PS-driven into the A-HIOT framework provide the researchers with a higher accuracy of finding optimized leads for a particular receptor with minimum set of false positives (FPs).

**Table 4:** Performance comparison of PS-driven DNNs framework with other ML algorithms: the comparison of benchmark performance of the PS-driven DNNs/DL framework for hit/lead optimization employing PS module

Algorithm	Dataset	Accuracy	Sensitivity	Specificity	AUC-ROC
Random Forest (RF)	Internal test set (*x'*)^a^	0.802	0.754	0.821	0.801
	Small independent validation dataset^b^	0.614	0.724	0.488	0.822
	Large independent benchmark dataset^c^	0.726	0.817	0.827	0.834
Extreme Gradient Boost (XGB)	Internal test set (*x'*)^a^	0.806	0.786	0.813	0.812
	Small independent validation dataset^b^	0.631	0.763	0.534	0.699
	Large independent benchmark dataset^c^	0.782	0.838	0.621	0.848
Deep Neural Networks (DNNs/DL)	Internal test set (*x'*)^a^	0.818	0.913	0.824	0.812
	Small independent validation dataset^b^	0.859	0.872	0.822	0.884
	Large independent benchmark dataset^c^	0.899	0.902	0.924	0.898

^a^Total of 175 (81 inhibitors and 94 non-inhibitors) partitioned into 7:3 classified as Internal test set (*x'*)

^b^46 (35 inhibitors and 11 non-inhibitors) classified as small independent validation dataset

^c^1886 (86 inhibitors and 1800 decoys (termed as non-inhibitors)) classified as large independent benchmark dataset

### Performance of A-HIOT for hit/lead identification and optimization on androgen receptor (AR): case study

To ascertain the hit identification and optimization competency of A-HIOT for any receptor, irrelevant to CXCR4, we chose AR for a separate case study. The AR is a type of nuclear receptor, also known as nuclear receptor subfamily 3, group C, member 4 (NR3C4) and is activated by testosterone. The AR participate significantly in prostate cancer thus anti-androgens used to treat the same.

**Fig. 9:**
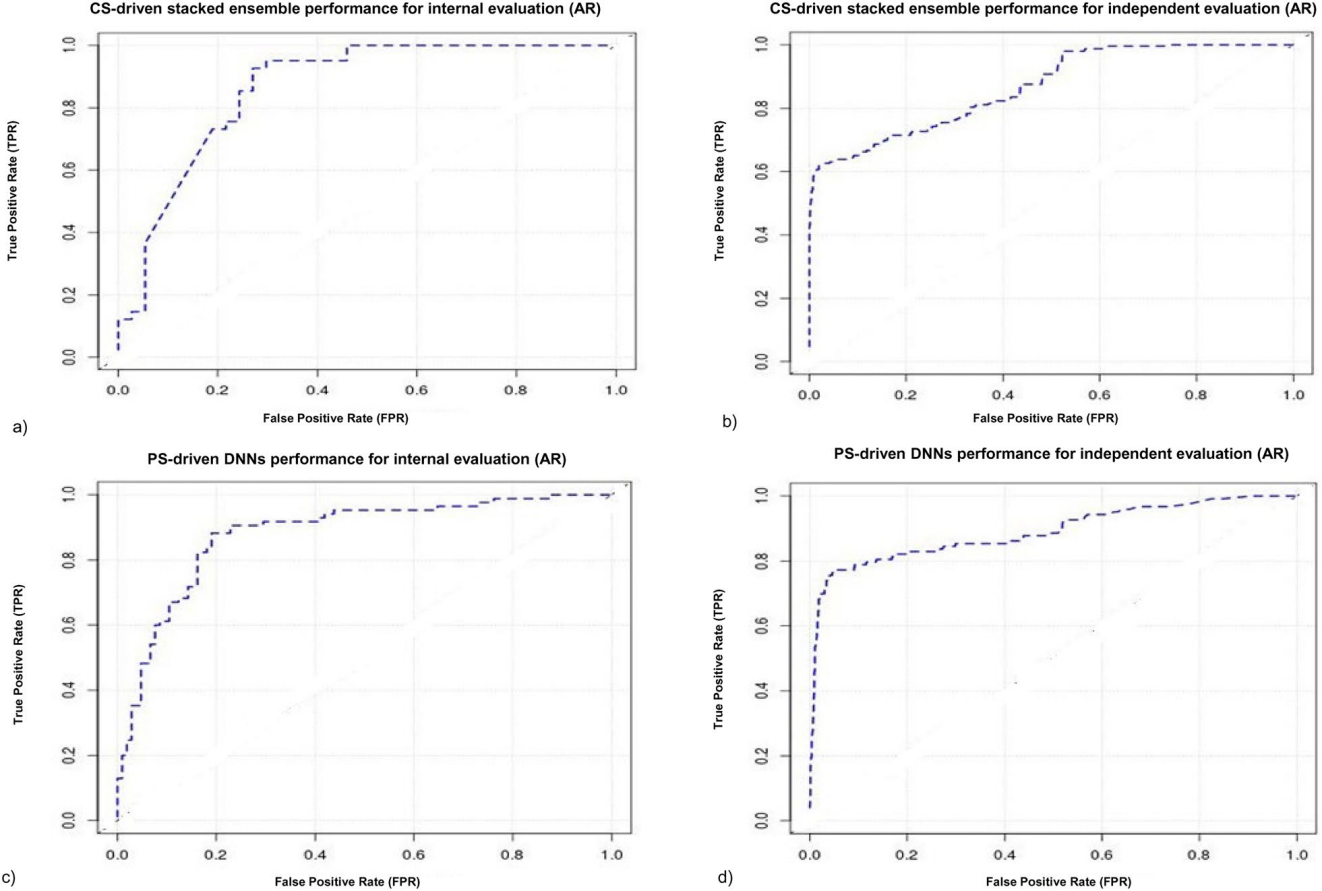
The performance comparison of A-HIOT on AR: case study. The internal evaluation was performed employing random split of training dataset into 7:3 ratios and AUC-ROC plot (**a**) demonstrates 86.4% training performance for CS-module and the independent test dataset obtained 86.8% AUC-ROC (**b**), that translates the satisfactory classification and hits/leads identification capacity. Similar approach was used in PS-module for internal evaluation wherein, the approach obtained 87.9% AUC-ROC shown in (**c**) and for independent test dataset its obtained 90.2% AUC-ROC shown in (**d**). The exceptional performance if PS-module dictates the power of A-HIOT for hits/leads optimization

The training of CS-module of A-HIOT demonstrated satisfactory classification performance by achieving 86.4% AUC (Fig. 9a, b) along with balanced 0.845 sensitivity and 0.902 specificity. In addition, the 0.867 accuracy of trained model. In comparison with classification and hits/leads identification power from test dataset, the CS-module of A-HIOT achieved 86.8% AUC, 0.892 sensitivity and 0.886 specificity and 0.882 accuracy and shown in Table 5. The sensitivity (measure of identifying hits/leads (TPs)) and specificity (measure of eliminating false hits/leads (TNs)) translates the hits/leads identification power of CS-module of A-HIOT. The CS- module identified 126 active and 752 inactive molecules and comprises the dataset input for PS-module of A-HIOT.

**Table 5:** Performance Comparison of CS- and PS- modules of A-HIOT on androgen receptor (AR): the performance comparison of the CS- and PS- modules of A-HIOT for internal and independent evaluation for optimized hits/leads using androgen receptor

Algorithm	Dataset	Accuracy	Sensitivity	Specificity	AUC-ROC
CS-module					
CS-driven Stacked Ensemble	Internal test set (*x'*)^a^	0.867	0.845	0.902	0.864
	Independent test dataset^b^	0.882	0.892	0.886	0.868
PS-module					
PS-driven Deep Neural Networks (DNNs/DL)	Internal test set (*x'*)*	0.852	0.823	0.894	0.879
	Independent test dataset^c^	0.919	0.862	0.924	0.902

^a^Total of 303 (146 inhibitors and 157 non-inhibitors) partitioned into 7:3 classified as Internal test set (*x'*) for both CS- and PS-modules

^b^1121 (249 inhibitors and 872 non-inhibitors) classified as independent test dataset for CS-module

^c^878 (126 inhibitors and 752 non-inhibitors) classified as small independent validation dataset

The training dataset for PS-module comprise of 303 molecules and test dataset has 878 molecules. The ML-ready dataset prepared as per PS-module protocol by calculating protein–ligand interaction Klekota-Roth substructure fingerprint count (binary fingerprints). The training of PS-module achieved 87.9% AUC along with 0.823 sensitivity and 0.894 specificity. The trained model obtained 0.852 accuracy that dictates the effectiveness of entire model. In comparison when trained model applied to test dataset and obtained 90.2% AUC, 0.862 sensitivity, 0.924 specificity, and 0.919 accuracy (Fig. 9c, d). The PS-module reported higher AUC and specificity scores that defines the sufficiency of A-HIOT for correct elimination of decoys or false hit and retrieval of true optimized hit. The TPs were further extracted and concatenated along with protein–ligand interaction profile scores and ranked as per interaction threshold (6–8). We retrieved eight optimized hit/lead molecules.

## Conclusion

VS is a supremely in-demand technique to find potential drug-like molecules from ultra-large virtual libraries for the desired target. While finding potential molecules, there are chances of substantial false hits and lack of biological selectivity for the desired target, which is expensive and time-consuming. The existing VS algorithms identify hits or lead that further demand optimization for in vitro examination. Therefore, we attempted to develop a novel method that simultaneously identifies and optimizes hit/lead molecules by integrating chemical- and protein-space-driven architectures and stands for an automated-hit identification and optimization tool (A-HIOT).

Benchmarking and case study for AR experiments show that the performance of both CS and PS modules of A-HIOT are superior to several other individual ML/DNN frameworks when assessed on the benchmark dataset for family of GPCR receptors (CXCR4 and AA2AR) and androgen receptor (AR). The attractive advantages of our A-HIOT framework can be reflected in the following aspects. First, the CS-driven stacked ensemble framework does not inherit probabilistic nature allows it to effectively explore the feature space to obtain the best accuracy and specificity of the predictive model that can identify hits (TPs) required for receptor target. Second, the PS-driven DNNs framework, which learns from fingerprint information and picks up specifically well interacting molecules (TPs) as per substructure fingerprint count presence. Third, the TPs produced by PS-driven DNN framework were combined with PLIP of TPs as per the fixed threshold that are in line with the pharmacophore hypothesis and selected as optimized hits.

The A-HIOT can be considered as a generalized framework that will implement to find novel active molecules or the drug-repositioning task. While on assessing the family of GPCR receptors, A-HIOT generated a list of the optimized hits/leads mixture of inhibitor molecules with higher specificity and AUC that were found to be active against CXCR4 and AA2AR receptors. Thus, A-HIOT serves the purpose of finding new drug-like molecules as well re-positioned molecules active for other receptors and to demonstrate this aspect, we carried out an independent case study by using AR. Being an independent case study, the A-HIOT performed exceptionally well for finding optimized leads. The optimized hits/leads can directly go to in vitro experiments that reduce the cost and time of lead optimization and HTS. The A-HIOT brings chemical and protein spaces together, bridging a long-standing gap between the respective fields. The pipeline caters to chemists and biologists and compels them to confidently execute a VS or drug-repurposing task, even if computational awareness is low. We assume that integrating AI, framework streamlining, and human intervention reduction can boost in silico drug discovery and repositioning.

## Supplementary Information

**Additional file 1**. Supplementary Section.

**Additional file 2: Fig S1**. Random Forest (RF) classification performance. The AUC-ROC plots illustrate the augmented classification performance achieved by RF algorithm when implemented individually. Initially, the RF trained employing standard dataset that obtained 99.42% training (a) and 89.10% for internal evaluation (b) set. The algorithm obtained 99.07% (c) and 80.72% (d) AUC-ROC plots representing training and prediction for small independent validation dataset and 99.51% (f), 82.3% (g) for large independent benchmark dataset. The instances used to train and benchmark RF algorithm presented in (e and h).

**Additional file 3: Fig. S2.** Extreme Gradient Boost (XGB) classification performance. The AUC-ROC plots illustrate the augmented classification performance achieved by XGB algorithm when implemented individually. Initially, the XGB trained employing standard dataset that obtained 99.93% training (a) and 81.2% for internal evaluation (b) set. The algorithm obtained 99.81% (c) and 78.2% (d) AUC-ROC plots representing training and prediction for small independent validation and 99.78% (f) and 78.7% (g) for large independent benchmark dataset. The instances used to train and benchmark XGB algorithm presented in (e and h).

**Additional file 4: Fig. S3**. Deep Neural Network/Deep Learning (DNN/DL) classification performance. The AUC-ROC plots illustrate the augmented classification performance achieved by DNN/DL algorithm when implemented individually. Initially, the DNN/DL trained employing standard dataset that obtained 99.63% training (a) and 91.4% for internal test (b) set. The algorithm obtained 99.81% (c) and 86.62% (d) AUC-ROC plots representing training and prediction for small independent validation and 99.78% (f) and 95.1 (g) for large independent benchmark dataset. The instances used to train DNN/DL algorithm presented in (e and h).

**Additional file 5: Fig. S4**. Optimized hits retrieved via CS-driven stacked ensemble from independent dataset. The stacked ensemble identified 35 hit molecules. Herein, the DNN-driven predictive model concatenated along with PLIP score procedure implemented for hit optimization and we found four molecules and showcased in this figure.

**Additional file 6: Table S1**. Chemical composition of training dataset.

**Additional file 7: Table S2**. Details of feature descriptors used in present study to create feature space(ɸ).

**Additional file 8: Table S3**. Details of class of feature instances used in RF prognostic model construction.

**Additional file 9: Table S4**. Details of class of feature instances used in XGB prognostic model construction.

**Additional file 10: Table S5**. Details of class of feature instances used in DNNs/DL predictive model construction.

**Additional file 11: Table S6**. Details of feature descriptors used for model development and validation.

## Data Availability

The A-HIOT is available at https://gitlab.com/neeraj-24/A-HIOT for details. The training and independent datasets are available on repository.
