# Supplementary material for: Machine intelligence-driven framework for optimized hit selection in virtual screening
Source: J Cheminform. 2022 Jul 22;14:48. doi: 10.1186/s13321-022-00630-7 (PMC9306080; doi:10.1186/s13321-022-00630-7)
Supplement: Supplementary file 1 — Additional file 1. Supplementary Section. [file 13321_2022_630_MOESM1_ESM.docx]

**Supplementary Section**

**Machine intelligence-driven framework for optimized hit selection in virtual screening**

Neeraj Kumar^1,2^, Vishal Acharya^1,2,^ *

^1^Functional Genomics and Complex System Lab, Biotechnology Division, CSIR-Institute of Himalayan Bioresource Technology, Palampur 176061, Himachal Pradesh, India

^2^Academy of Scientific and Innovative Research, Ghaziabad 201002, India

**Materials and Method**

**Machine learning (ML) models used for comparison with CS-stacked ensemble module**

We used random forest (RF), extreme gradient boost (XGB) and deep neural networks (DNNs) for performance comparison of CS-driven staked ensemble framework.

The standard notation is used to define the data and classifiers:

- *Ɗ* represents the training dataset comprising *N* (preprocessed feature vector dataset) known instances of input and response variables:

(1)

$$Ɗ= \left\{ \left( x_{n,}y_{n} \right),n=1,...N \right\}, x\epsilon\phi, y$$

Here, *x* is the input consisting of *D* feature vectors (molecular descriptors and fingerprints),$\phi is the feature space$, *y* is the coupled response variable, and *x_n_* represents the n-th feature vector of the instance.

- RF combines multiple decision trees and produces classification outcomes. The RF was implemented using the *randomForest* library. Initially, the RF was trained for 1500 trees, and a [*random subset of the features*](https://en.wikipedia.org/wiki/Random_subspace_method) was applied for the learning process to reduce overfitting. The training process was followed by RF tuning (*tRF*) to reduce the OOB error and to select the best model with improved RF parameters (*ntreeTry* = 1200, stepFactor = 1.5, improve = 0.0001 were the primary parameters for tuning). Gini impurity was considered as a measure of misclassification and would act when a randomly chosen sample of a node of the test set is classified randomly based on labels assigned to the node.

$$G_{k}\left( n \right)=1-\sum_{i=1}^{C} p\left( i \right)*\left( 1-p\left( i \right) \right)$$

(2)

where,$G_{k}\left( n \right) is the$Gini impurity for node *n*, *C* is the class number, and $p\left( i \right)$ is the probability of a randomly chosen sample for element *i*. Automatic feature selection and plotting were performed for the RF, and a set of top 30 features was selected.

- XGB, a scalable tree boosting and supervised ML algorithm, was implemented using the *xgboost* library in the R platform. The XGB follows an "additive strategy" for training the ML model. For a given training dataset sample *n* with representative feature vectors *x_n_* and response variable *y_n_*, the predicted output using *K* additive function is given as follows:

$$ŷ_{n}=1-\sum_{k=1}^{K} f_{k}\left( x_{n} \right), f_{k}\epsilon F$$

(3)

where *f_k_* constitutes tree structures for *x_n_* and *F* comprises all classification trees built for the predicted results. The *xgb.cv* (cross-validation) module was implemented to determine the best parameters that would yield the best accuracy upon five-fold cross-validation. The optimized parameters receive input for training at *nrounds* = 1500 iterations using *binary:logistic* objective and *gbtree* booster with a cross-validation output for internal validation of the model (*eval_metric*). To achieve an optimized model, iterations were performed using a fixed partition of the data. At each step, the performance was evaluated using a separate held-out validation set.

- The DL (MLP) architecture was adapted using the *H2O* library in R. The initial training dataset $Ɗ$ was received within the input layer. The MLP network was composed of four hidden-layer units (600, 300, 200, and 2). The first three layers used "*Tanh*" activation function, and the fourth layer included an output unit composed of two nodes which is beneficial for binary classification. The grid search was used for the optimization of hyperparameters to obtain a model with the best accuracy and classification, which is an array of two sets, with the first and second sets consisting of three (600, 200, 300) and two (300, 2) hidden-layer units, respectively; moreover, the first three layers of each set used the "*Tanh*" activation function. To prune overfitting, dropout (*input_dropout_ratio* and *rate* parameters) with a rate of 0.2 was used. The grid search framed the best model with optimized parameters and was used for further training and testing purposes.

**Model validation**

The A-HIOT framework’s validation was performed using a small and a benchmark independent datasets. The feature vector calculation step was followed by validation and benchmarking dataset preparation where dataset was extracted as per training dataset feature vectors. The entire validation and benchmark process was conducted as per training approach. Model validation was evaluated using sensitivity, specificity, AUC, and the rate of correctly classified molecules.

**Training parameter details**

**Table 1**: Details of training parameters used in stacked ensemble

| **Parameter** | **Value** |
| --- | --- |
| **Base Layer: Extreme Gradient Boost (XGB)** | |
| Training_frame | train |
| ntrees | 1500 |
| max_depth | 5 |
| min_rows | 2 |
| learn_rate | 0.2 |
| nfolds | 10 |
| fold_assignment | Random |
| keep_cross_validation_predictions | TRUE |
| **Base Layer: Random Forest (RF)** | |
| Training_frame | train |
| ntrees | 1500 |
| max_depth | 5 |
| nfolds | 10 |
| fold_assignment | Random |
| keep_cross_validation_predictions | TRUE |
| **Super Learner or Meta Layer: Deep Neural Networks (DNNs)** | |
| 1. **Grid search hyperparameters (stack_param)** | |
| epochs | 50 |
| Hidden | c(400,200,2) |
| score_interval | 1 |
| stopping_rounds | 3 |
| stopping_metric | “AUC” |
| 1. **Model parameters** | |
| training_frame | train |
| base_models | list(my_rf, my_xgb) |
| metalearner_algorithm | deeplearning |
| metalearner_params | stack_param |
