# Supplementary material for: Machine intelligence-driven framework for optimized hit selection in virtual screening
Source: J Cheminform. 2022 Jul 22;14:48. doi: 10.1186/s13321-022-00630-7 (PMC9306080; doi:10.1186/s13321-022-00630-7)
Supplement: Supplementary file 2 — Additional file 2: Fig S1. Random Forest (RF) classification performance. The AUC-ROC plots illustrate the augmented classification performance achieved by RF algorithm when implemented individually. Initially, the RF trained employing standard dataset that obtained 99.42% training (a) and 89.10% for internal evaluation (b) set. The algorithm obtained 99.07% (c) and 80.72% (d) AUC-ROC plots representing training and prediction for small independent validation dataset and 99.51% (f), 82.3% (g) for large independent benchmark dataset. The instances used to train and benchmark RF algorithm presented in (e and h). [file 13321_2022_630_MOESM2_ESM.pptx]

## Slide 1
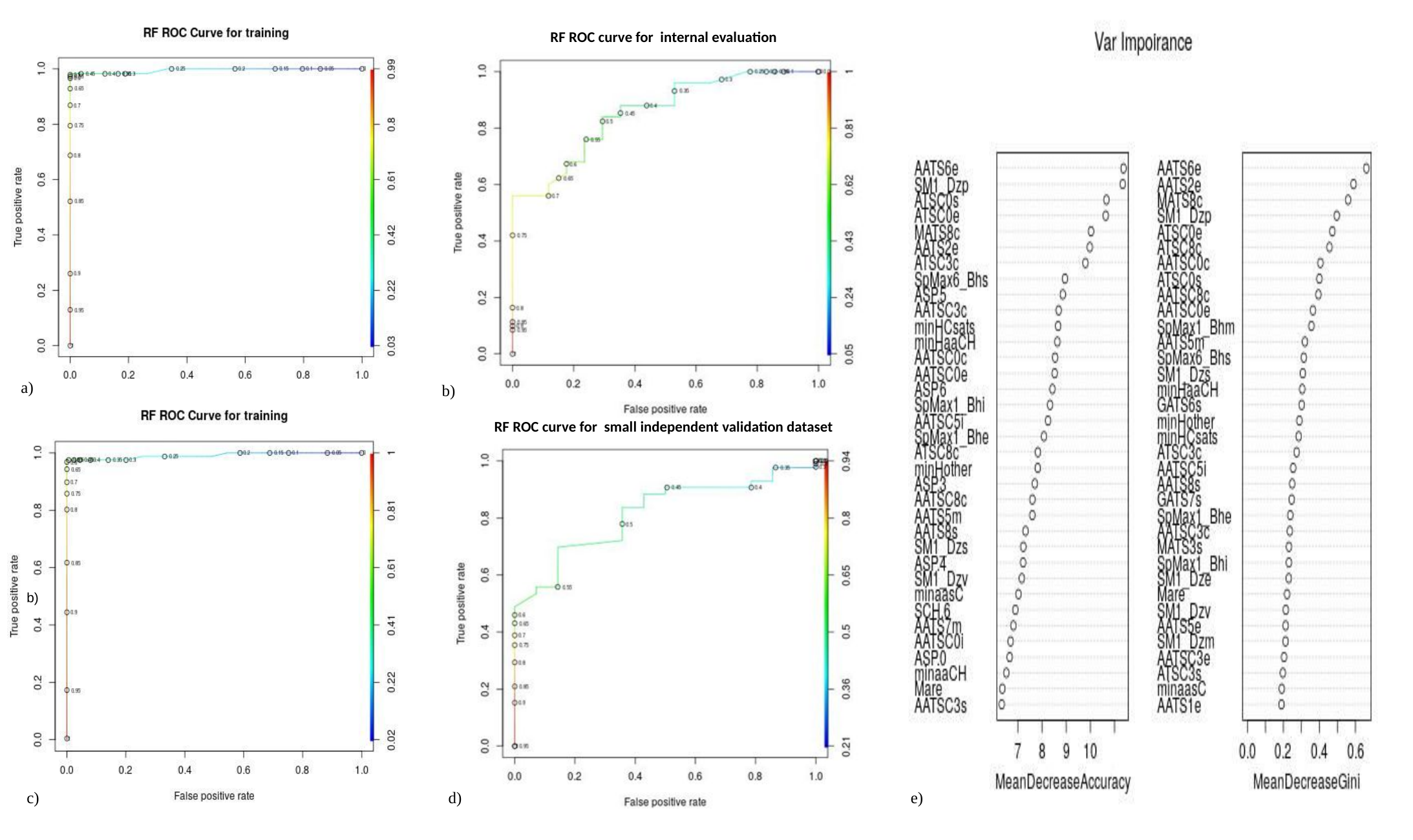

b)
a)
b)
c)
d)
e)
RF ROC curve for internal evaluation
b)
RF ROC curve for small independent validation dataset
d)

## Slide 2
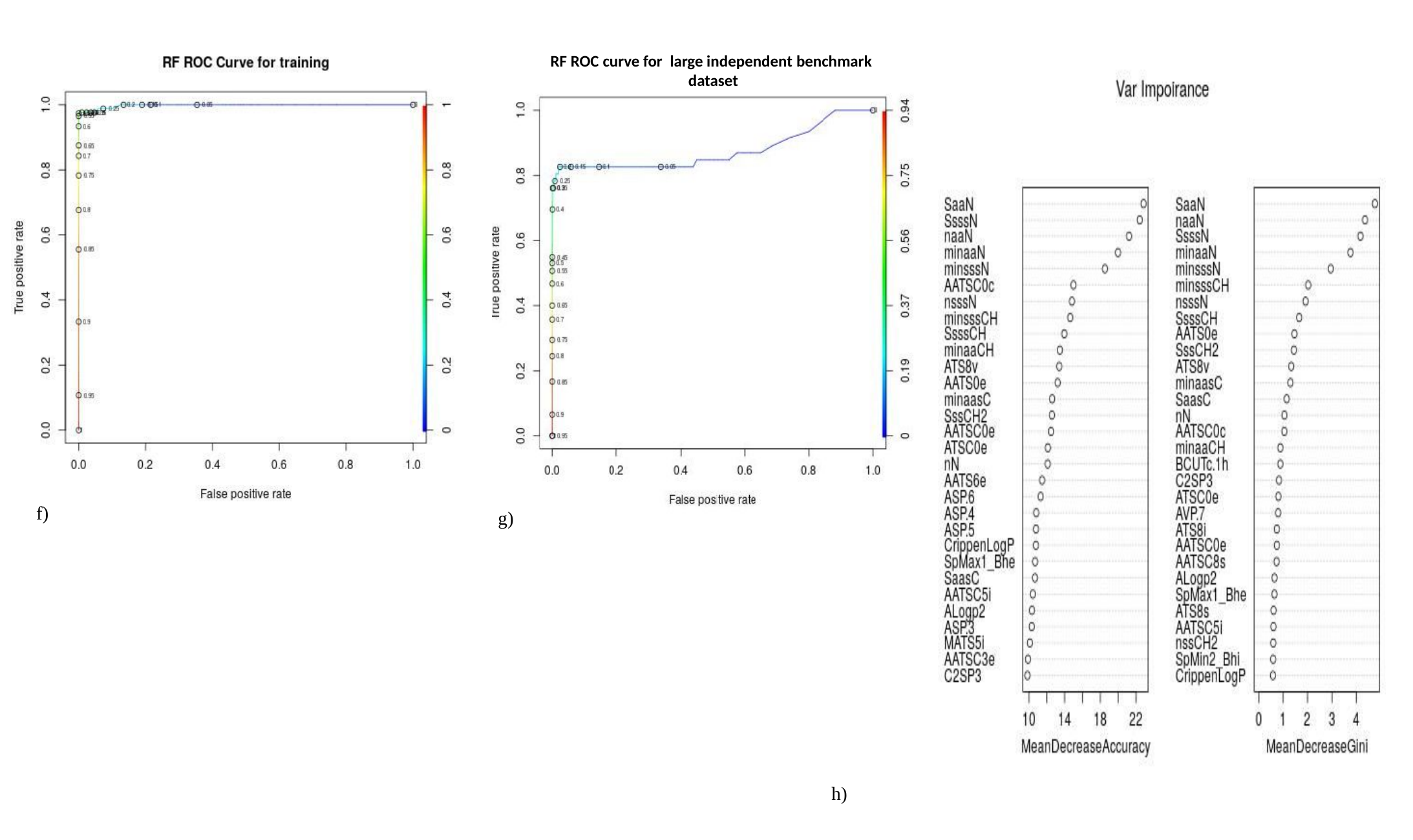

RF ROC curve for large independent benchmark
 dataset
f)
g)
h)
