# Supplementary material for: Machine intelligence-driven framework for optimized hit selection in virtual screening
Source: J Cheminform. 2022 Jul 22;14:48. doi: 10.1186/s13321-022-00630-7 (PMC9306080; doi:10.1186/s13321-022-00630-7)
Supplement: Supplementary file 3 — Additional file 3: Fig. S2. Extreme Gradient Boost (XGB) classification performance. The AUC-ROC plots illustrate the augmented classification performance achieved by XGB algorithm when implemented individually. Initially, the XGB trained employing standard dataset that obtained 99.93% training (a) and 81.2% for internal evaluation (b) set. The algorithm obtained 99.81% (c) and 78.2% (d) AUC-ROC plots representing training and prediction for small independent validation and 99.78% (f) and 78.7% (g) for large independent benchmark dataset. The instances used to train and benchmark XGB algorithm presented in (e and h). [file 13321_2022_630_MOESM3_ESM.pptx]

## Slide 1
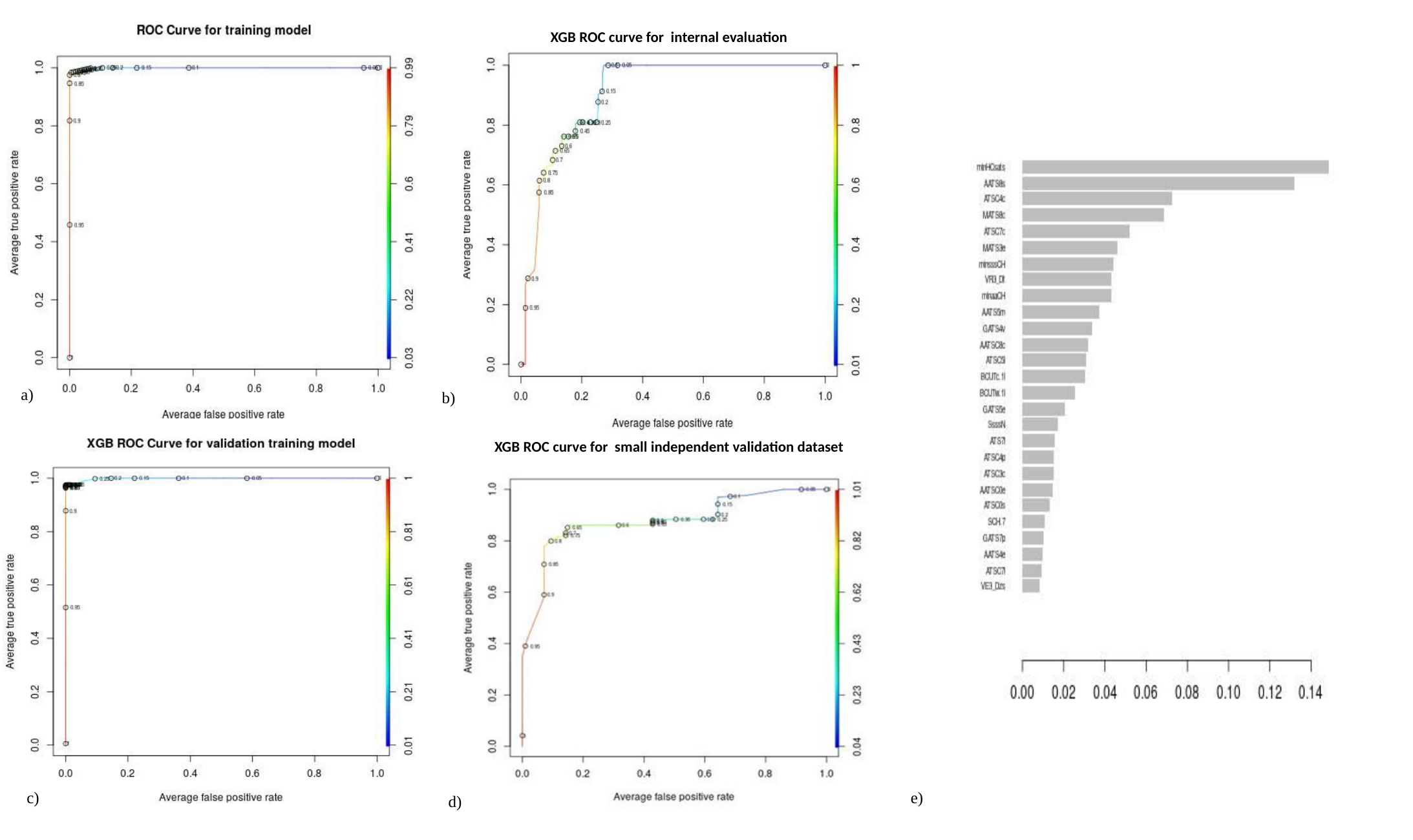

a)
b)
c)
d)
e)
XGB ROC curve for internal evaluation
b)
XGB ROC curve for small independent validation dataset
d)

## Slide 2
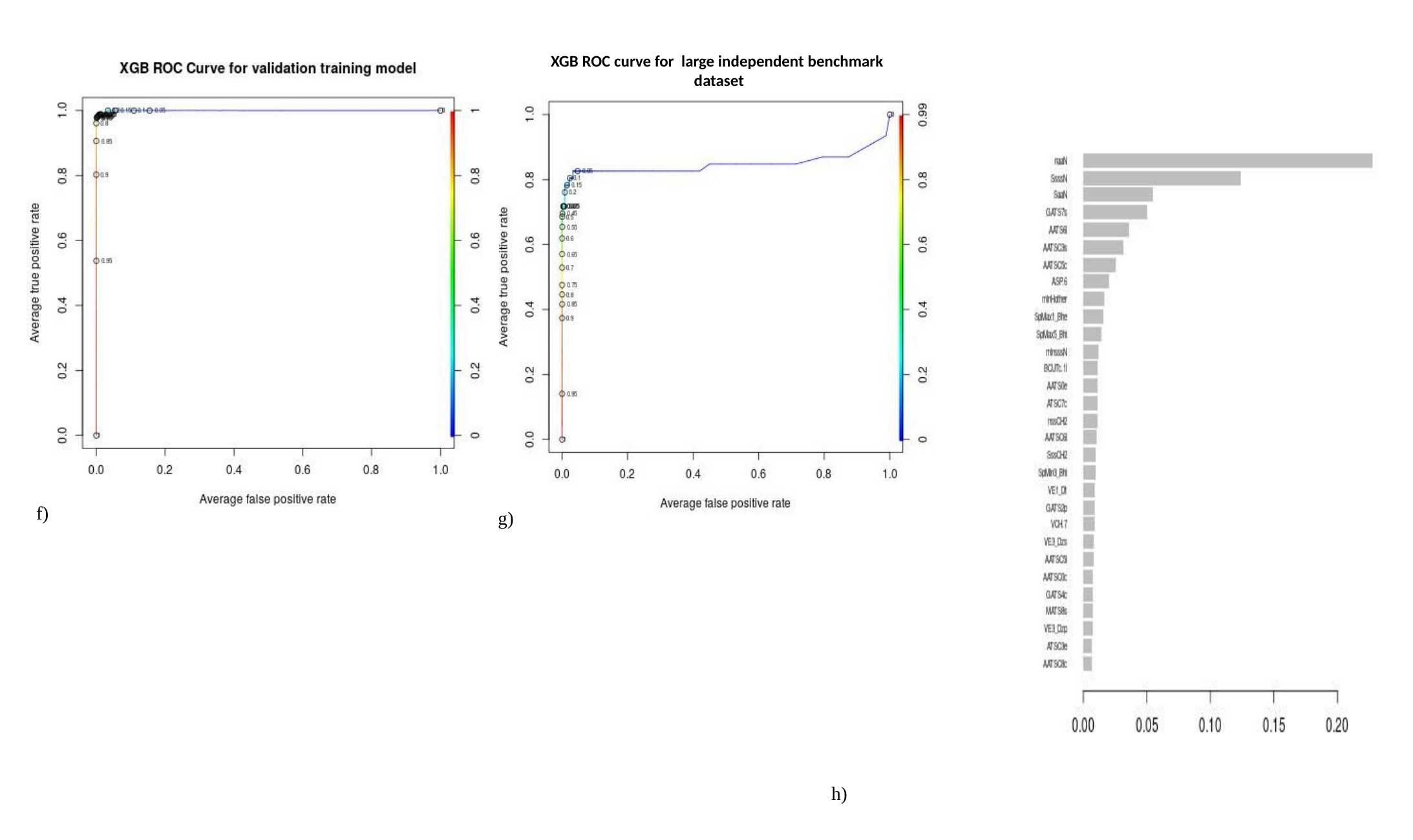

XGB ROC curve for large independent benchmark
 dataset
f)
g)
h)
