# Supplementary material for: Machine intelligence-driven framework for optimized hit selection in virtual screening
Source: J Cheminform. 2022 Jul 22;14:48. doi: 10.1186/s13321-022-00630-7 (PMC9306080; doi:10.1186/s13321-022-00630-7)
Supplement: Supplementary file 4 — Additional file 4: Fig. S3. Deep Neural Network/Deep Learning (DNN/DL) classification performance. The AUC-ROC plots illustrate the augmented classification performance achieved by DNN/DL algorithm when implemented individually. Initially, the DNN/DL trained employing standard dataset that obtained 99.63% training (a) and 91.4% for internal test (b) set. The algorithm obtained 99.81% (c) and 86.62% (d) AUC-ROC plots representing training and prediction for small independent validation and 99.78% (f) and 95.1 (g) for large independent benchmark dataset. The instances used to train DNN/DL algorithm presented in (e and h). [file 13321_2022_630_MOESM4_ESM.pptx]

## Slide 1
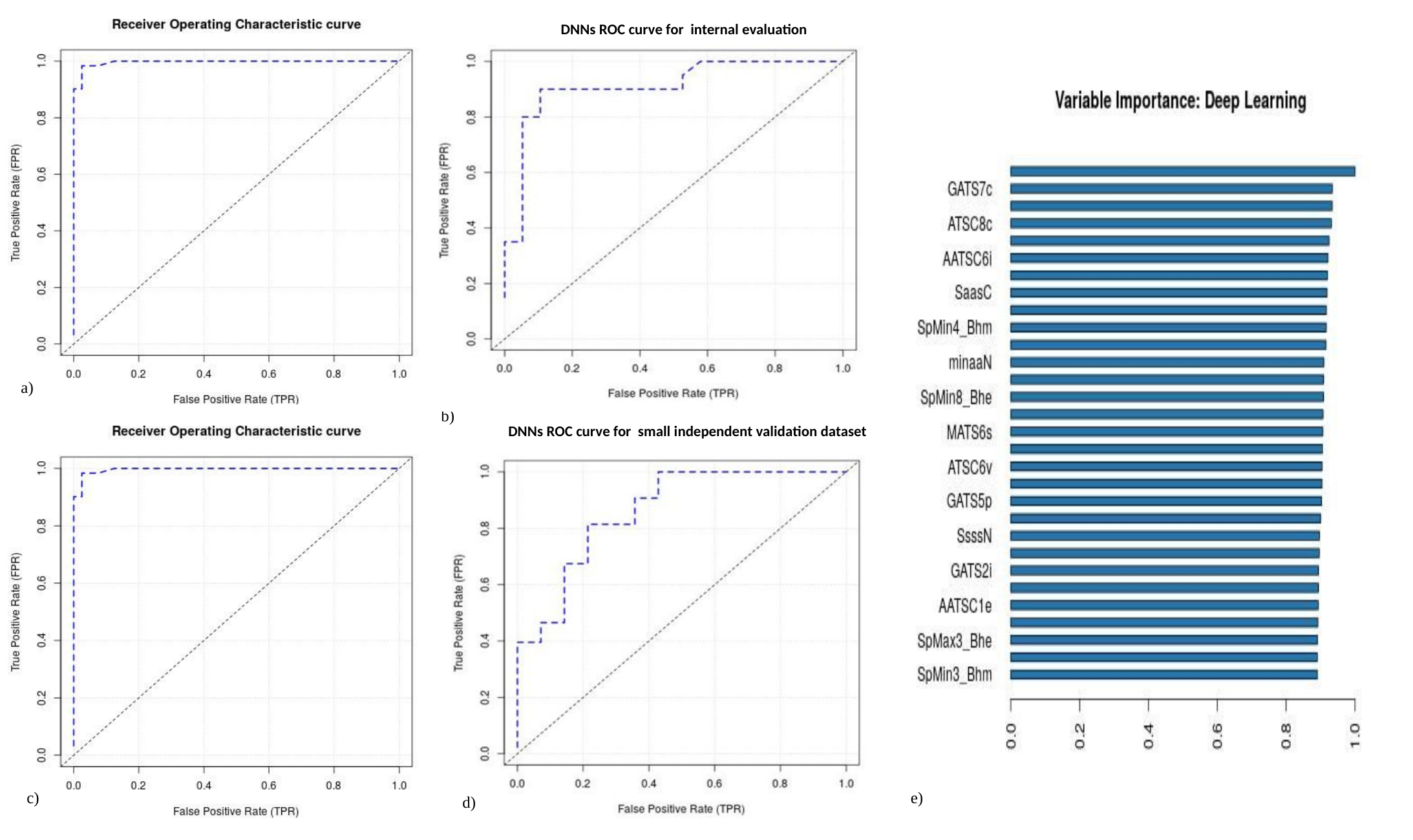

a)
b)b)
c)
d)
e)
DNNs ROC curve for internal evaluation
DNNs ROC curve for small independent validation dataset
d)

## Slide 2
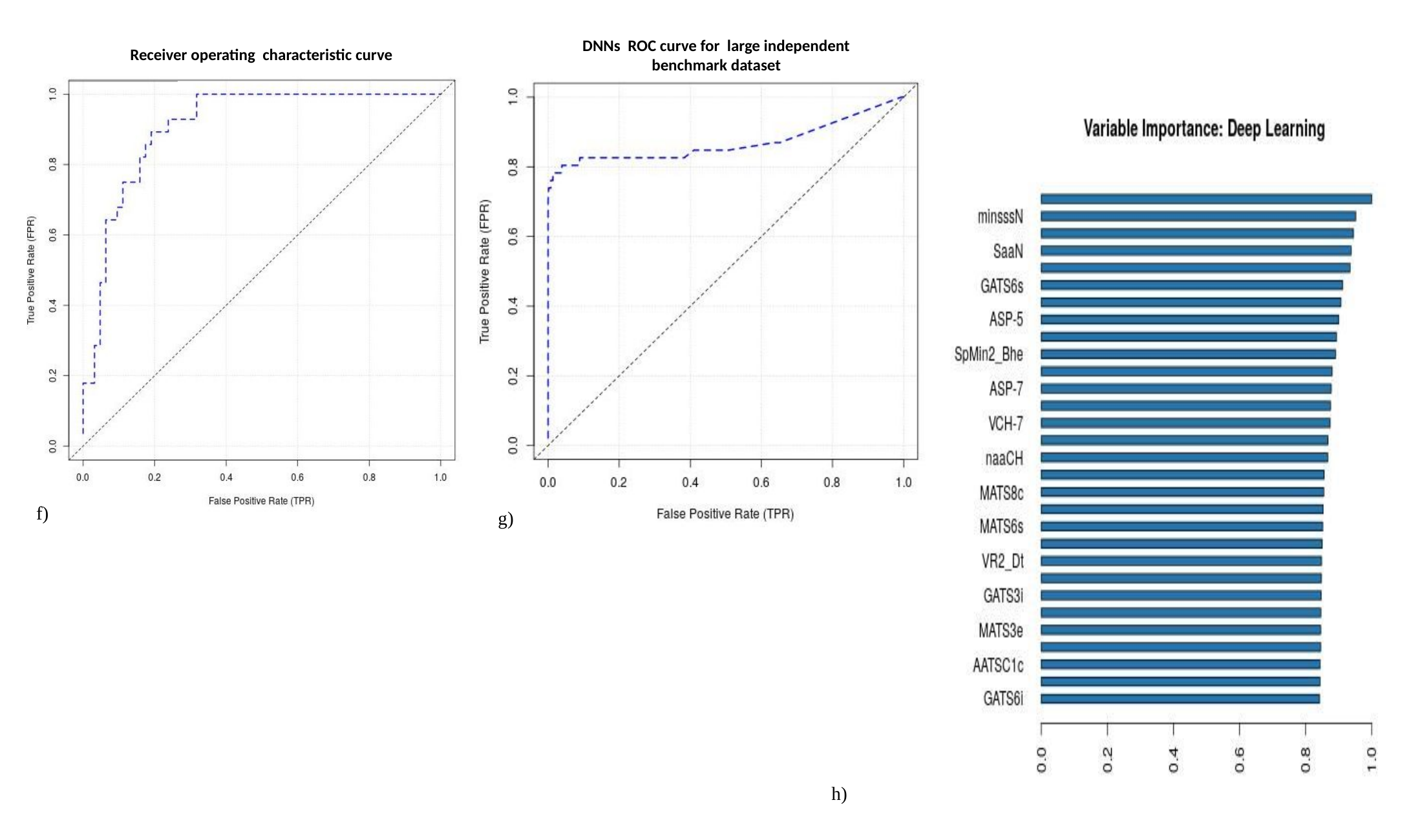

DNNs ROC curve for large independent benchmark dataset
Receiver operating characteristic curve
f)
g)
h)
