# Supplementary figures and images for: Machine intelligence-driven framework for optimized hit selection in virtual screening
Source: J Cheminform. 2022 Jul 22;14:48. doi: 10.1186/s13321-022-00630-7 (PMC9306080; doi:10.1186/s13321-022-00630-7)

## Slide 1
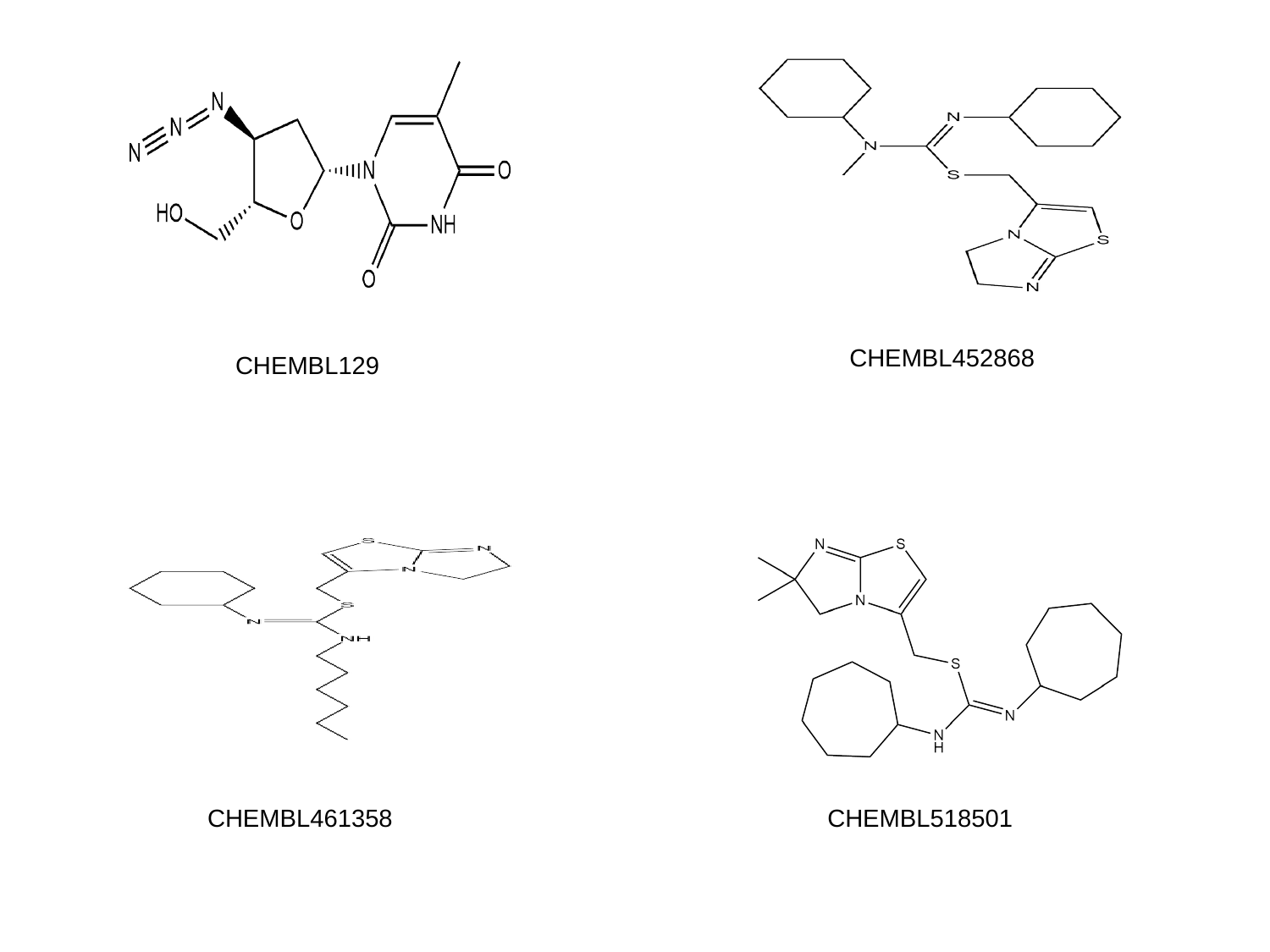

CHEMBL452868
CHEMBL129
CHEMBL461358
CHEMBL518501

Supplement: Supplementary file 5 — Additional file 5: Fig. S4. Optimized hits retrieved via CS-driven stacked ensemble from independent dataset. The stacked ensemble identified 35 hit molecules. Herein, the DNN-driven predictive model concatenated along with PLIP score procedure implemented for hit optimization and we found four molecules and showcased in this figure. [file 13321_2022_630_MOESM5_ESM.pptx]
