# Supplementary material for: Machine intelligence-driven framework for optimized hit selection in virtual screening
Source: J Cheminform. 2022 Jul 22;14:48. doi: 10.1186/s13321-022-00630-7 (PMC9306080; doi:10.1186/s13321-022-00630-7)
Supplement: Supplementary file 6 — Additional file 6: Table S1. Chemical composition of training dataset. [file 13321_2022_630_MOESM6_ESM.docx]

**Supplementary Table 1:** Chemical composition of training dataset.

| **Family** | **Number of compounds** | **Reference** |
| --- | --- | --- |
| Cyclic peptides | 4 | [1] |
| Tetrahydroquinoline derivatives | 110 | [2] |
| Indole derivatives | 7 | [2] |
| AMD derivatives | 15 | [3] |
| Macrocyclic polyamines | 4 | [4] |
| Cyclic ring system | 35 | [5] |

**Reference**

[1] Tanaka T, Tsutsumi H, Nomura W, Tanabe Y, Ohashi N, Esaka A, et al. Structure-activity relationship study of CXCR4 antagonists bearing the cyclic pentapeptide scaffold: identification of the new pharmacophore. Org Biomol Chem 2008;6:4374–7. https://doi.org/10.1039/B812029C.

[2] Debnath B, Xu S, Grande F, Garofalo A, Neamati N. Small molecule inhibitors of CXCR4. Theranostics 2013;3:47–75. https://doi.org/10.7150/THNO.5376.

[3] Përez-Nueno VI, Ritchie DW, Rabal O, Pascual R, Borrella JI, Teixidó J. Comparison of ligand-based and receptor-based virtual screening of HIV entry inhibitors for the CXCR4 and CCR5 receptors using 3D ligand shape matching and ligand-receptor docking. J Chem Inf Model 2008;48:509–33. https://doi.org/10.1021/ci700415g.

[4] Rusconi S, Cicero M Lo, Viganò O, Sirianni F, Bulgheroni E, Ferramosca S, et al. New Macrocyclic Amines Showing Activity as HIV Entry Inhibitors Against Wild Type and Multi-Drug Resistant Viruses. Mol 2009, Vol 14, Pages 1927-1937 2009;14:1927–37. https://doi.org/10.3390/MOLECULES14051927.

[5] Mishra RK, Shum AK, Platanias LC, Miller RJ, Schiltz GE. Discovery and characterization of novel small-molecule CXCR4 receptor agonists and antagonists. Sci Reports 2016 61 2016;6:1–9. https://doi.org/10.1038/srep30155.
